# CHL1 depletion affects dopamine receptor D2-dependent modulation of mouse behavior

**DOI:** 10.3389/fnbeh.2023.1288509

**Published:** 2023-11-09

**Authors:** Luciana Fernandes, Ralf Kleene, Ludovica Congiu, Sandra Freitag, Matthias Kneussel, Gabriele Loers, Melitta Schachner

**Affiliations:** ^1^Zentrum für Molekulare Neurobiologie, Universitätsklinikum Hamburg-Eppendorf, Hamburg, Germany; ^2^Institut für Molekulare Neurogenetik, Zentrum für Molekulare Neurobiologie Hamburg, ZMNH, Universitätsklinikum Hamburg-Eppendorf, Hamburg, Germany; ^3^Department of Cell Biology and Neuroscience, Keck Center for Collaborative Neuroscience, Rutgers University, Piscataway, NJ, United States

**Keywords:** close homolog of L1, CHL1, dopamine, dopamine receptor D2, behavior, quinpirole, sulpiride

## Abstract

**Introduction:**

The dopaminergic system plays a key role in the appropriate functioning of the central nervous system, where it is essential for emotional balance, arousal, reward, and motor control. The cell adhesion molecule close homolog of L1 (CHL1) contributes to dopaminergic system development, and CHL1 and the dopamine receptor D2 (D2R) are associated with mental disorders like schizophrenia, addiction, autism spectrum disorder and depression.

**Methods:**

Here, we investigated how the interplay between CHL1 and D2R affects the behavior of young adult male and female wild-type (CHL+/+) and CHL1-deficient (CHL1−/−) mice, when D2R agonist quinpirole and antagonist sulpiride are applied.

**Results:**

Low doses of quinpirole (0.02 mg/kg body weight) induced hypolocomotion of CHL1+/+ and CHL1−/− males and females, but led to a delayed response in CHL1−/− mice. Sulpiride (1 mg/kg body weight) affected locomotion of CHL1−/− females and social interaction of CHL1+/+ females as well as social interactions of CHL1−/− and CHL1+/+ males. Quinpirole increased novelty-seeking behavior of CHL1−/− males compared to CHL1+/+ males. Vehicle-treated CHL1−/− males and females showed enhanced working memory and reduced stress-related behavior.

**Discussion:**

We propose that CHL1 regulates D2R-dependent functions *in vivo*. Deficiency of CHL1 leads to abnormal locomotor activity and emotionality, and to sex-dependent behavioral differences.

## 1. Introduction

Cells of the dopaminergic system localize in the ventral tegmental area and substantia nigra of the midbrain and control many central and peripheral nervous system functions. Brain regions are innervated by the three major dopaminergic pathways, the nigrostriatal, the mesocorticolimbic, and the tuberoinfundibular pathways. These dopaminergic pathways control a multitude of functions, such as movement, cognition, decision-making, motivation, working memory, mood, emotions, balanced visceral motility, secretion of hormones, positive reinforcement, and pain (Björklund and Dunnett, 2007; Iversen and Iversen, 2007; Klein et al., 2019; Donovan and Wood, 2022; Kouhnavardi et al., 2022; Vegas-Suarez et al., 2022). Dopamine (DA) is not only a neurotransmitter modulating nervous system functions, but also an important immunoregulatory factor affecting both innate and adaptive immunity (Levite et al., 2017; Pinoli et al., 2017; Matt and Gaskill, 2020; Vidal and Pacheco, 2020). Impaired dopaminergic (DAergic) signaling is implicated in several neurological and psychiatric disorders, such as Parkinson’s disease, autism spectrum disorder, attention deficit/hyperactivity disorder, depression, schizophrenia, drug abuse, and neuroinflammation (Arias-Carrión and Pŏppel, 2007; Goto and Grace, 2007; Sierra et al., 2015; Belujon and Grace, 2017; Kosillo and Bateup, 2021; Mariggio et al., 2021; Guatteo et al., 2022; Garritsen et al., 2023). DAergic neurons of the midbrain are the main source of DA in the mammalian central nervous system, where DA is packed into synaptic vesicles and released into the synaptic cleft upon activation (Meiser et al., 2013; Garritsen et al., 2023). DA serves its function by activating five subtypes of receptors (D1-D5), which are abundant in the brain. They are classified into two general classes: those that predominantly couple to the Gαs/olf class of G proteins (“D1-like”; D1 and D5 receptors), and to the Gαi/o class of G proteins (“D2-like”; D2-D4 receptors) (Kebabian and Calne, 1979; Lachowicz and Sibley, 1997; Kim, 2023). The D2 receptor (D2R) is expressed pre- and postsynaptically and maintains continuous signal transduction through agonist-induced receptor phosphorylation, arrestin recruitment, and endocytosis, which recycle and resensitize desensitized receptors (Kim, 2023). Activation of the D2Rs and interaction with Gαi/o inhibits the activation of adenylyl cyclase, production of cyclic adenosine monophosphate and activation of protein kinase A (Neve et al., 2004; Beaulieu and Gainetdinov, 2011). In mammals, D2R is localized both on pre- and postsynaptic dopaminergic neurons. Two splice variants of D2R are found to display differences in DA binding affinity, trafficking and induction of signaling pathways. The short D2R isoform is primarily localized at the presynaptic membrane of DAergic neurons where it acts as an autoreceptor to reduce DA synthesis and release, resulting in initial inhibitory control over locomotion at low agonist doses (Usiello et al., 2000; Radl et al., 2018). The long D2R isoform, predominantly expressed postsynaptically in striatal medium spiny neurons, acts as an auto- or hetero-receptor and induces motor activity upon activation by DA or agonists (Dal Toso et al., 1989; David et al., 1991; Usiello et al., 2000; Ford, 2014; Shioda, 2017; Radl et al., 2018; Quintana and Beaulieu, 2019). Both D2R isoforms play key roles in the DAergic system and do not function properly in schizophrenia (Oda et al., 2015; Shioda, 2017; Zhou et al., 2022; Iasevoli et al., 2023). The DAergic system regulates locomotion (Beninger, 1983; Groenewegen, 2003), motivational aspects of behavior like exploration, novelty and reward seeking (Menegas et al., 2017) as well as anxiety (Steiner et al., 1997; Brañdao and Coimbra, 2019; Juza et al., 2023). Antagonists and partial agonists of D2Rs are used to treat schizophrenia, autism spectrum disorder, Parkinson’s disease, depression, and anxiety (Mandic-Maravic et al., 2022; Juza et al., 2023). Furthermore, DA receptors play crucial roles in signaling upon novelty stimuli (Kakade and Dayan, 2002) and mediate hippocampus-dependent memory consolidation of novel events (Düzel et al., 2010).

We have identified CHL1 as binding partner and signaling modulator of D2Rs (Kotarska et al., 2020). CHL1 is a member of the immunoglobulin superfamily, which is mainly present at the axon and accumulates at synapses (Nikonenko et al., 2006; Dityatev et al., 2008; Andreyeva et al., 2010). CHL1 is involved in neurite outgrowth, cell migration, positioning, and survival and regulates dendritic spine density (Hillenbrand et al., 1999; Buhusi et al., 2003; Montag-Sallaz et al., 2003; Demyanenko et al., 2004; Nishimune et al., 2005; Jakovcevski et al., 2009; Mohan et al., 2019). CHL1 was found to interact with both D2R isoforms, to inhibit the internalization of the short D2R isoform and to regulate development of ventral midbrain DAergic pathways (Alsanie et al., 2017; Kotarska et al., 2020). Moreover, CHL1 is implicated in mental retardation and psychiatric disorders, such as schizophrenia and autism spectrum disorders (Sakurai et al., 2002; Frints et al., 2003; Chen et al., 2005; Tam et al., 2010; Salyakina et al., 2011; Shoukier et al., 2013). The CHL1 gene is located on chromosome 3 at 3p26.1, a region which contains genes that are suggested to be associated with schizophrenia (Lewis et al., 2003; Gandawijaya et al., 2020). Deletions in the 3p region result in 3p-syndrome which is characterized by developmental delay, intellectual disability, facial dysmorphisms, and autism-spectrum disorder behaviors (Gandawijaya et al., 2020; Fu et al., 2021). CHL1 deficiency in mice leads to age-dependent loss of parvalbumin-expressing hippocampal interneurons, impairments in synaptic transmission, long-term potentiation, working memory, gating of sensorimotor information, and prepulse inhibition of the acoustic startle response (Montag-Sallaz et al., 2003; Irintchev et al., 2004; Nikonenko et al., 2006; Schmalbach et al., 2015). Conditional ablation of CHL1 in neurons and constitutive ablation of CHL1 in mice lead to alterations in social and exploratory behaviors (Montag-Sallaz et al., 2002, 2003; Pratte et al., 2003; Irintchev et al., 2004; Morellini et al., 2007; Kolata et al., 2008; Pratte and Jamon, 2009).

Since CHL1 interacts with D2Rs and reduces internalization of the short D2R isoform, CHL1−/− mice may contain less functional D2Rs at the cell membrane and thus be less responsive to D2R autoreceptor stimulation. To test this hypothesis, we determined D2R-dependent mouse behavior. We evaluated the performance of male and female CHL1-deficient (CHL1−/−) and wild-type (CHL1+/+) mice treated with low doses of the D2R agonist quinpirole and the D2R antagonist sulpiride, which target mainly presynaptic D2 autoreceptors, in different behavioral paradigms. Based on our results from the open field, Y-maze, novel object and social interaction, we propose that CHL1 regulates D2R-dependent functions, thereby affecting behavior in sex-dependent and sex-independent manners.

## 2. Materials and methods

### 2.1. Chemicals

(−)-Quinpirole hydrochloride [(−)-LY-171555 or trans-(−)-(4aR)-4,4a,5,6,7,8,8a,9-octahydro-5-propyl-1H-pyrazolo[3,4-g]quinoline monohydrochloride; CAS number 85798–08-9] was obtained from Tocris-Bioscience (catalog #1061, lot 17A/239398; Wiesbaden-Nordenstadt, Germany). (±)-Sulpiride [(±)-5-(aminosulfonyl)-N-[(1-ethyl-2-pyrrolidinyl)methyl]-2-methoxybenzamide; CAS number 15676–16-1] (catalog #S8010, lot 4B/217248) and dimethyl sulfoxide (CAS number 67–68-5) were from Sigma-Aldrich (Taufkirchen, Germany).

### 2.2. Animals

Heterozygous CHL1-deficient mice back-crossed onto the C57Bl/6 J background for more than eight generations were used to obtain CHL1−/− mice and age-matched CHL1+/+ littermates (Montag-Sallaz et al., 2002). Mice were bred and maintained at the Universitätsklinikum Hamburg-Eppendorf. Animals were housed at 21 ± 1°C and 40–50% humidity on a 12 h light/12 h dark cycle with *ad libitum* access to food and water. Three- to five-month-old males or females were used for all experiments. Experiments were approved by the Behörde für Justiz und Verbraucherschutz of the State of Hamburg (animal permit number N061/2019) and experiments were designed and the manuscript was prepared according to the ARRIVE guidelines (Kilkenny et al., 2010).

### 2.3. Behavior

**Figure 1:**
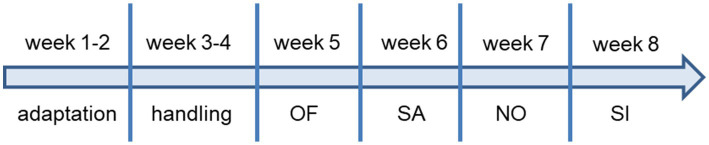
Timeline of behavioral experiments. OF, open field; SA, spontaneous alternation; NO, novel object; SI, social interaction. All experiments were performed with the same batch of mice and mice were 3-month-old when the experiments (OF) started and 4–5-month-old at the end of the experiments (SI).

To determine motor activity, exploratory behavior, and anxiety-like or stress-related behavior (Prut and Belzung, 2003; Seibenhener and Wooten, 2015) the open field test was used. Short-term memory retention, particularly spatial working memory and spontaneous alternation were tested in a free-trial Y-maze (Lalonde, 2002; Kraeuter et al., 2019). Novelty-seeking behavior triggered by a new stimulus, for which there was no pre-existing recognition memory, was assessed in the novel object test (Barto et al., 2013; Akiti et al., 2022) and motivation to investigate a social stimulus was analyzed by giving the experimental mouse the choice to investigate an unfamiliar mouse or a familiar sex-matched mouse (Freitag et al., 2003). Tracks representing the position of the mice were created and analyzed with EthoVision XT (Noldus, Wageningen, The Netherlands; https://www.noldus.com/ethovision; RRID:SCR_000441) (Freitag et al., 2003). Manual scoring of grooming and jumping behavior was performed by a trained experimenter blinded to the genotype and treatment of the mice using The Observer software (Noldus). Between tests, mice were allowed to recover for 7 days before the next test with injection of a single dose of the solutions followed by behavioral evaluation after 2 min. All experiments were performed with the same batch of mice and with the same instruments. A detailed description of the tests is given in the Supplementary material and the timeline of experiments is shown in Figure 1.

### 2.4. Statistical analysis

A detailed description is given in the Supplementary material.

## 3. Results

### 3.1. Open field

Compared to CHL1+/+ mice, CHL1−/− mice exhibit a different exploratory behavior, a reduced reaction to novelty and impairments in their working memory (Montag-Sallaz et al., 2002, 2003; Pratte et al., 2003; Morellini et al., 2007; Pratte and Jamon, 2009). Novelty-seeking, exploratory behavior, anxiety and locomotor activity are also modulated by DA and the DA receptors (Beninger, 1983; Kakade and Dayan, 2002; Menegas et al., 2017; Brañdao and Coimbra, 2019; Juza et al., 2023). Thus, we set out to determine if CHL1 and D2R together influence exploratory behavior. CHL1+/+ and CHL1−/− mice were treated with vehicle solution, a low dose of the D2R agonist quinpirole (0.02 mg/kg body weight) or a low dose of the D2R antagonist sulpiride (1 mg/kg body weight) which act primarily on presynaptic D2Rs (Eilam and Szechtman, 1989; Van Hartesveldt et al., 1994; Boschen et al., 2011, 2015). Motor activity, exploratory behavior, stress-related and anxiety-like behavior were first evaluated in the open field test. Mice were injected with vehicle solution, quinpirole or sulpiride and placed in the open field 2 min after the injection to detect an early drug impact (Eilam and Szechtman, 1989; Frantz and Van Hartesveldt, 1995; Usiello et al., 2000; Anzalone et al., 2012; Lane et al., 2012). Representative tracks of CHL1+/+ and CHL1−/− vehicle-, sulpiride- and quinpirole-treated males and females are shown in Supplementary Figure S1.

#### 3.1.1. Alterations in locomotor activity

**Figure 2:**
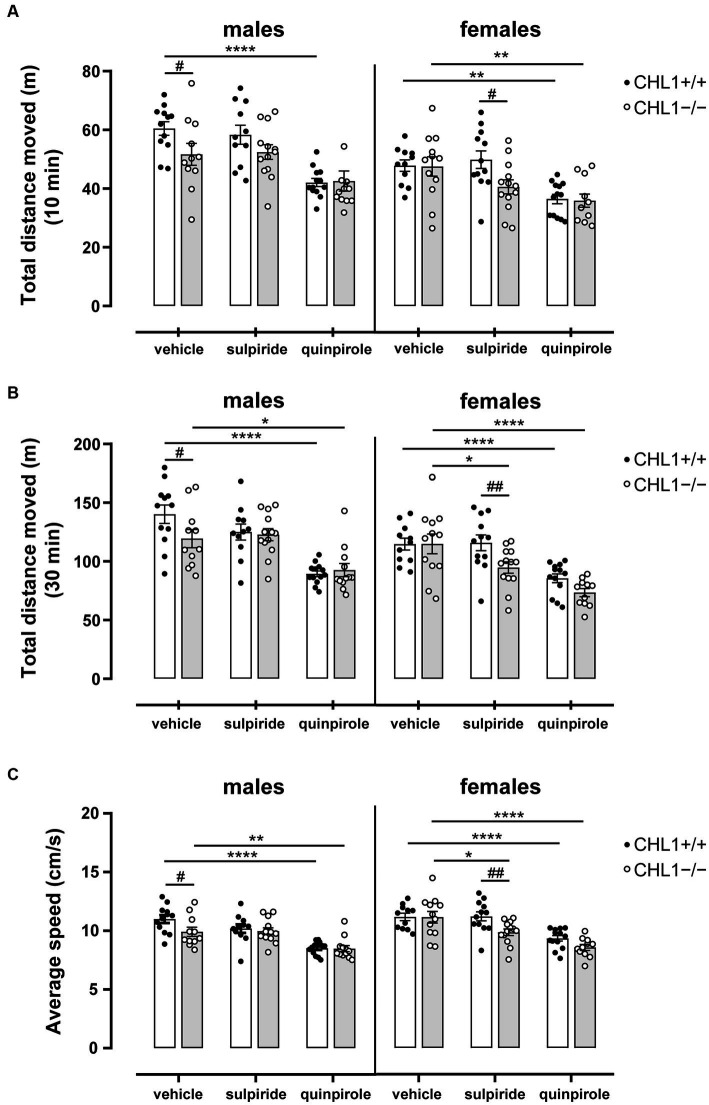
Reduced locomotor activity of male CHL1−/− mice in the open field and hypolocomotion of mice treated with quinpirole. Three-month-old male and female CHL1+/+ and CHL1−/− mice were treated with single injections of vehicle, sulpiride (1 mg/kg body weight) or quinpirole (0.02 mg/kg body weight), and activity in the open field was observed over 30 min. Total distance moved was evaluated after 10 min **(A)** and 30 min **(B)** in the open field and average speed over 30 min **(C)** was determined. Values are presented as single values and mean ± SEM (*n* = 11–13 mice per group) and were analyzed with three-way ANOVA (variables: genotype, treatment, and sex) followed by the Bonferroni correction *post-hoc* test (*p < 0.05, **p < 0.01, ****p < 0.0001, statistical difference from vehicle treated corresponding genotype; ^#^*p* < 0.05, ^##^*p* < 0.01 in case of genotype difference within treatment). White bars: CHL1+/+ mice, grey bars: CHL1−/− mice.

**Figure 3:**
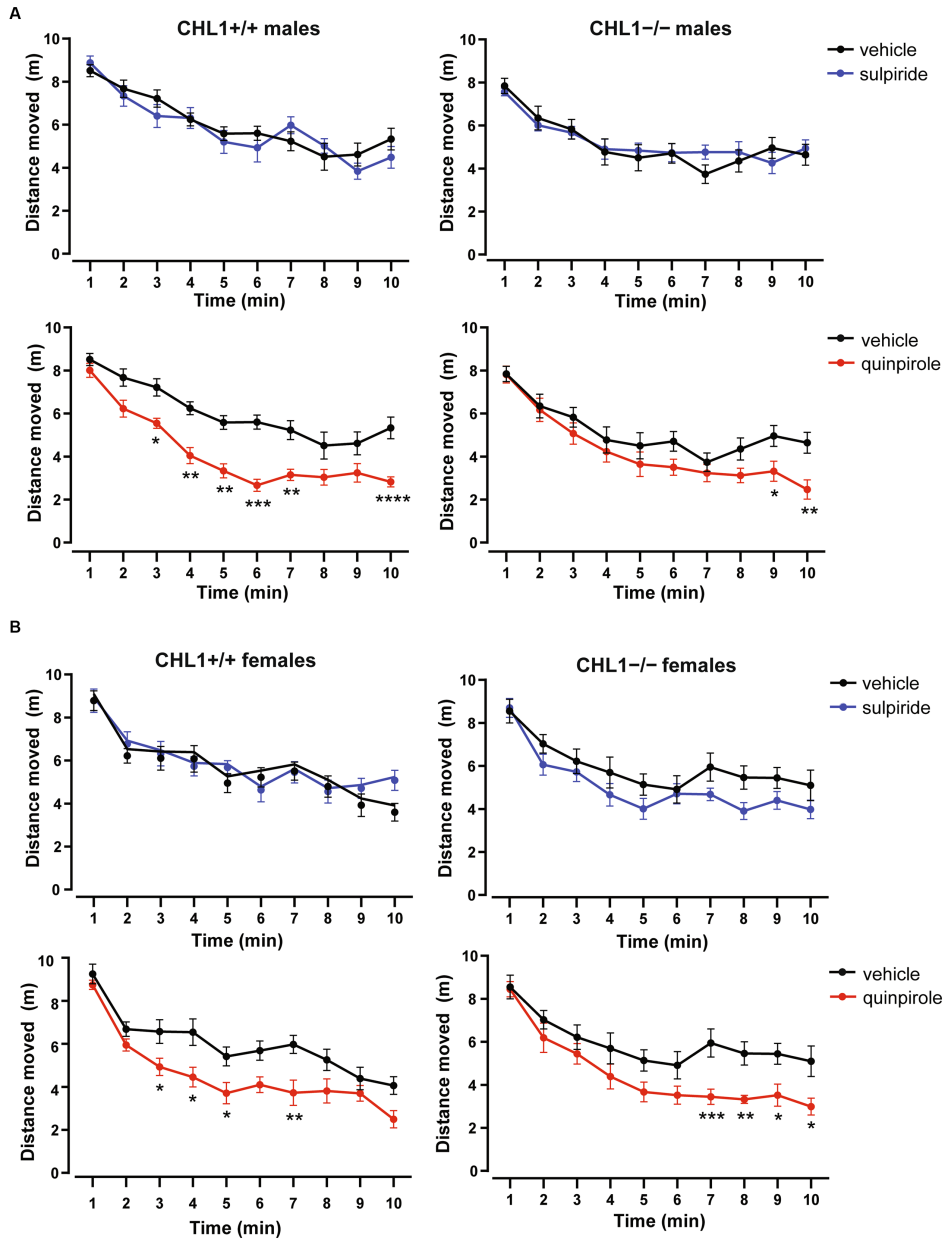
Delayed hypolocomotion of CHL1−/− mice treated with quinpirole. Three-month-old male **(A)** and female **(B)** CHL1+/+ and CHL1−/− mice were treated with single injections of vehicle, sulpiride (1 mg/kg body weight) or quinpirole (0.02 mg/kg body weight), and distance moved in the open field was observed for 10 min (comparison between genotypes is shown in Supplementary Figure S2). Values for each time bins are presented as mean ± SEM (*n* = 11–13 mice per group) and were analyzed with three-way repeated measures ANOVA followed by the Bonferroni correction *post-hoc* test (*p < 0.05, **p < 0.01, ***p < 0.001, ****p < 0.0001, statistical difference from vehicle treated corresponding genotype).

**Figure 4:**
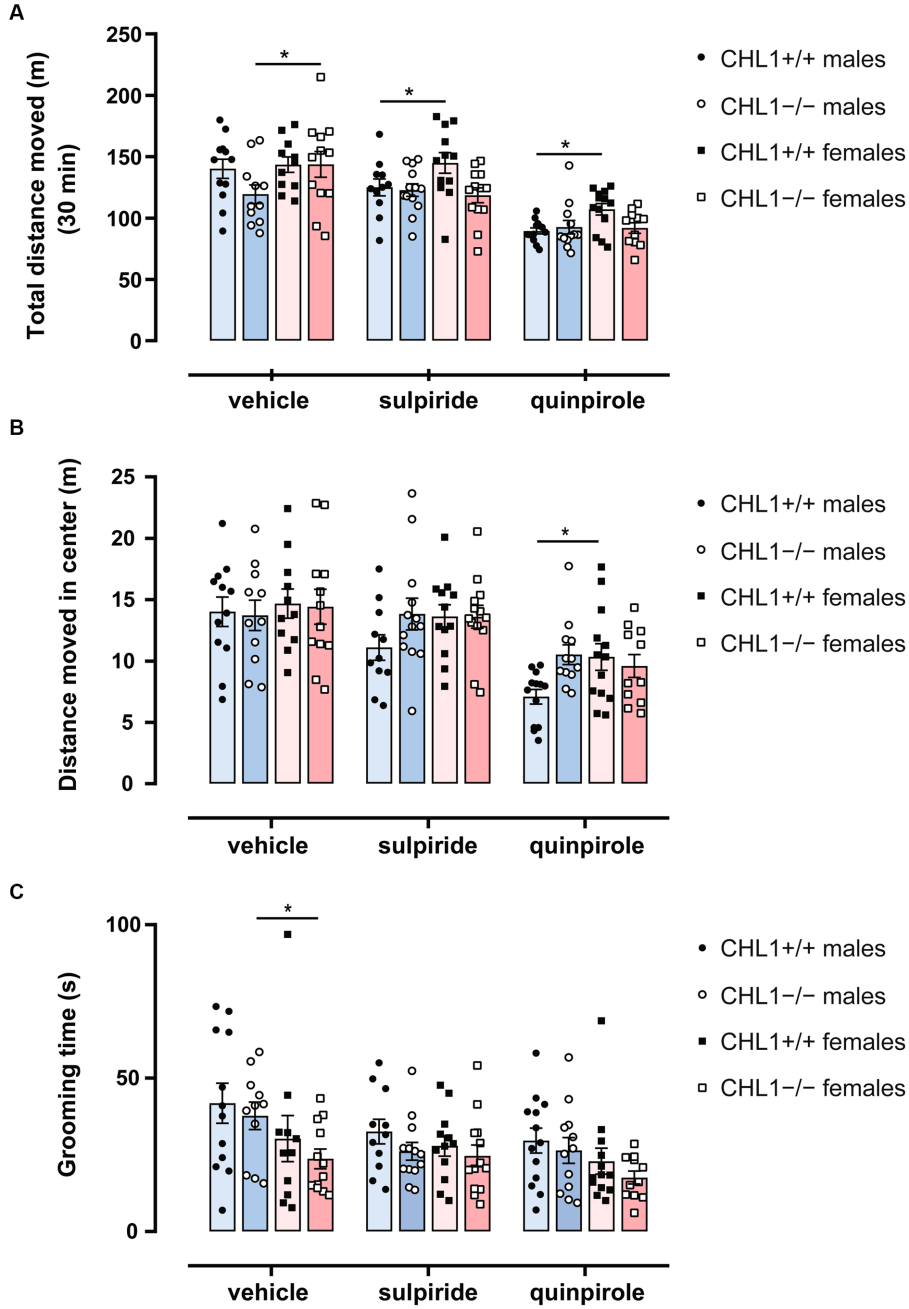
Sex-dependent differences in behavior in the open field. Three-month-old CHL1+/+ and CHL1−/− males and females were treated with single injections of vehicle, sulpiride (1 mg/kg body weight) or quinpirole (0.02 mg/kg body weight), and activity in the open field was observed over 30 min. After 30 min total distance moved **(A)**, distance moved in the center **(B)** and time spent grooming **(C)** were evaluated. The 30 min values are presented as single values and mean ± SEM (*n* = 11–13 mice per group) and were analyzed with three-way ANOVA (variables: genotype, treatment, and sex) followed by the Bonferroni correction *post-hoc* test (*p < 0.05, statistical difference for mice of the other sex but the same genotype and treatment). Blue bars: male mice, magenta bars: female mice.

Vehicle-treated CHL1−/− males moved a shorter distance and at a lower speed compared to the CHL1+/+ males treated with vehicle, while CHL1−/− and CHL1+/+ female mice treated with vehicle moved the same distance and at the same speed (Figures 2A–C, 3A,B, Supplementary Figure S2A). Interestingly, vehicle-injected CHL1−/− males also moved a shorter distance in comparison to CHL1−/− females (Figure 4A). Treatment with quinpirole reduced the distance moved of CHL1+/+ males within the first 10 min and differences to vehicle-treated CHL1+/+ males were already evident at 3 min (Figures 2A, 3A, Supplementary Figure S3A). In contrast, quinpirole led to reduced locomotion of CHL1−/− males only at 9 min and thereafter (Figures 2A,B, 3A, Supplementary Figure S3A). When all 30 min time points in the open field were evaluated, quinpirole injection led to a reduction in the distance moved and a reduction in average speed of CHL1−/− and CHL1+/+ males relative to vehicle-treated males, and quinpirole-treated CHL1−/− and CHL1+/+ males showed a similar distance moved and a comparable velocity (Figures 2B,C, Supplementary Figure S3A). CHL1+/+ males treated with quinpirole moved less compared to the quinpirole-treated CHL1+/+ females (Figures 3, 4A). Quinpirole reduced the distance moved of CHL1−/− and CHL1+/+ females within the first 10 min and in the overall 30 min of the test and values of quinpirole-treated CHL1+/+ and CHL1−/− females were similar (Figures 2A,B, 3B, Supplementary Figures S2C, S3B). When the first 10 min of the test were examined in detail CHL1+/+ females responded more quickly to quinpirole treatment and showed a reduction in the distance moved already after 3 min, while CHL1−/− females were delayed in responding to quinpirole and moved less at 7 min and at later times after application (Figure 3B). Also, quinpirole-treated CHL1+/+ males moved less compared to quinpirole-treated CHL1+/+ females, whereas quinpirole-treated CHL1−/− males and females moved similar distances (Figure 4A). Sulpiride treatment did not alter the activity of males in the open field and only reduced the distance moved and velocity of CHL1−/− females but not of CHL1+/+ females over 10 and 30 min (Figures 2A,B, 3A,B, 4A, Supplementary Figures S2B, S3A,B). In addition, sulpiride-treated CHL1+/+ males moved less compared to sulpiride-treated CHL1+/+ females, whereas sulpiride-treated CHL1−/− males and females moved similar distances (Figure 4A).

#### 3.1.2. Normal exploratory activity of CHL1−/− mice

**Figure 5:**
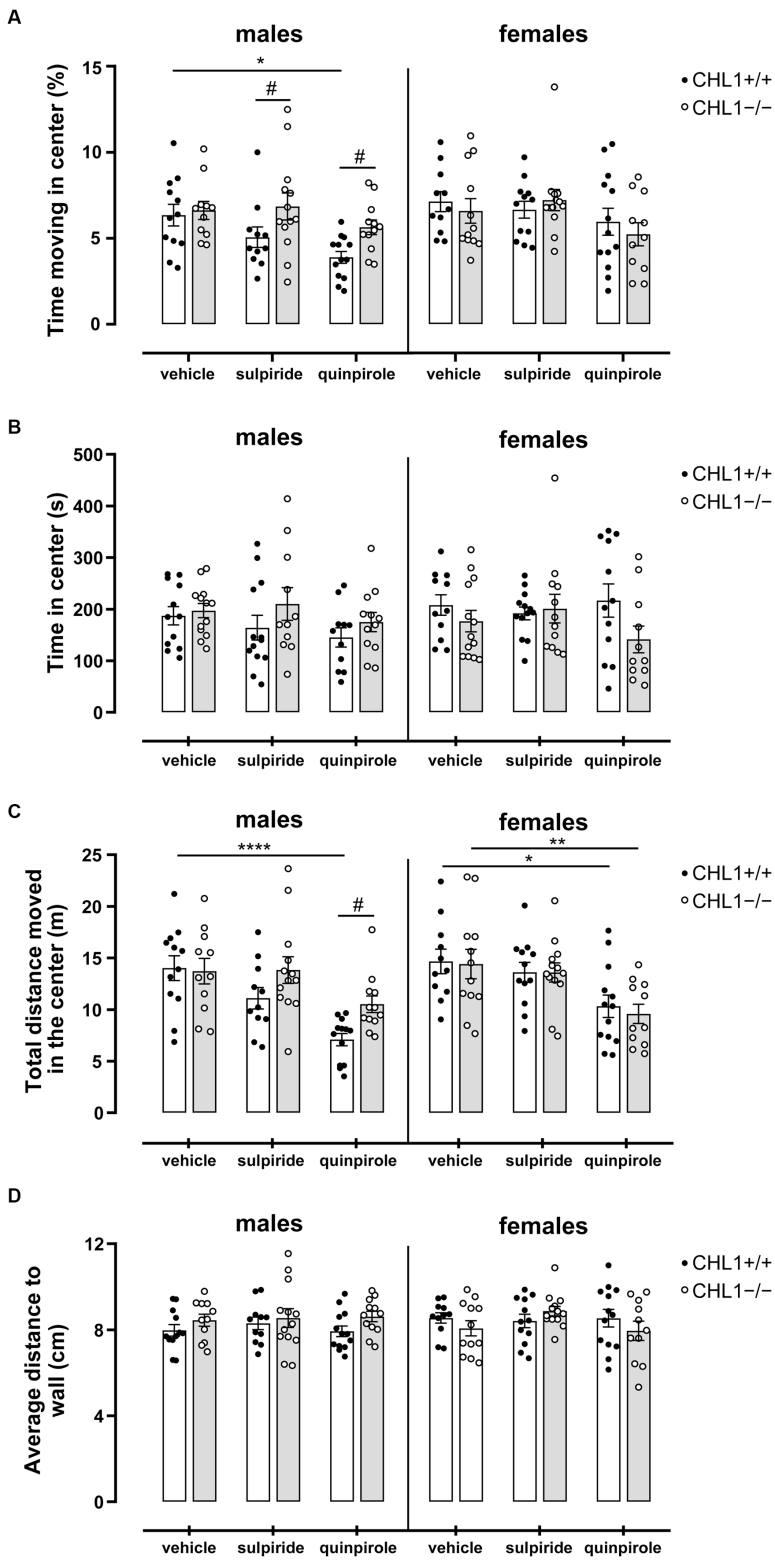
Normal stress-related behavior of CHL1−/− mice. Three-month-old CHL1+/+ and CHL1−/− males and females were treated with single injections of vehicle, sulpiride (1 mg/kg body weight) or quinpirole (0.02 mg/kg body weight), and activity in the open field was observed over 30 min. Time moving in the center **(A)**, time in center **(B)**, distance moved in the center (**C**; also shown in Figure 4B for sex comparisons) and average distance to wall **(D)** were determined. The 30 min values are presented as single values and mean ± SEM (*n* = 11–13 mice per group) and were analyzed with three-way ANOVA (variables: genotype, treatment and sex) followed by the Bonferroni correction *post-hoc* test (*p < 0.05, **p < 0.01, ****p < 0.0001, statistical difference from vehicle treated corresponding genotype; ^#^*p* < 0.05 in case of genotype difference within treatment). White bars: CHL1+/+ mice, grey bars: CHL1−/− mice.

Besides locomotor aptitude, exploratory patterns can give an indication of the emotional state and anxiety of the animal. Time and distance moved in the center zone and distance to wall can be used to evaluate the anxiety/stress-related behavior of mice (Seibenhener and Wooten, 2015). Time in the center zone revealed that vehicle-treated CHL1+/+ and CHL1−/− males and females spent a similar time moving in the center, stayed for comparable times in the center and moved similar total distances in the center zone (Figures 4B, 5A–C, Supplementary Figure S4). After injection, CHL1+/+ males, but neither CHL1−/− males nor CHL1+/+ and CHL1−/− females, showed a reduction in the time moving in the center or distance moved in the center. Quinpirole-treated CHL1+/+ males spent a shorter time in the center compared to quinpirole-treated CHL1+/+ females, while time in the center was similar for quinpirole-treated CHL1−/− males and females (Figure 5B, Supplementary Figure S4). Quinpirole-treated CHL1+/+ males moved a shorter distance in the center compared to the quinpirole-treated CHL1+/+ females (Figures 4B, 5C). Sulpiride-treated males and females of both genotypes did not differ from vehicle-treated mice in time and distance moved in the center, but sulpiride-treated CHL1+/+ males spent less time moving in the center compared to sulpiride-treated CHL1−/− males (Figures 4B, 5C). Average distances to wall were similar for vehicle-, sulpiride- and quinpirole-treated males and females of both genotypes (Figure 5D). These results suggest that CHL1−/− mice are not altered in their stress-related behavior and strengthen the finding that quinpirole treatment reduces locomotion especially of CHL1+/+ and CHL1−/− males.

#### 3.1.3. Altered emotional state of CHL1−/− males

**Figure 6:**
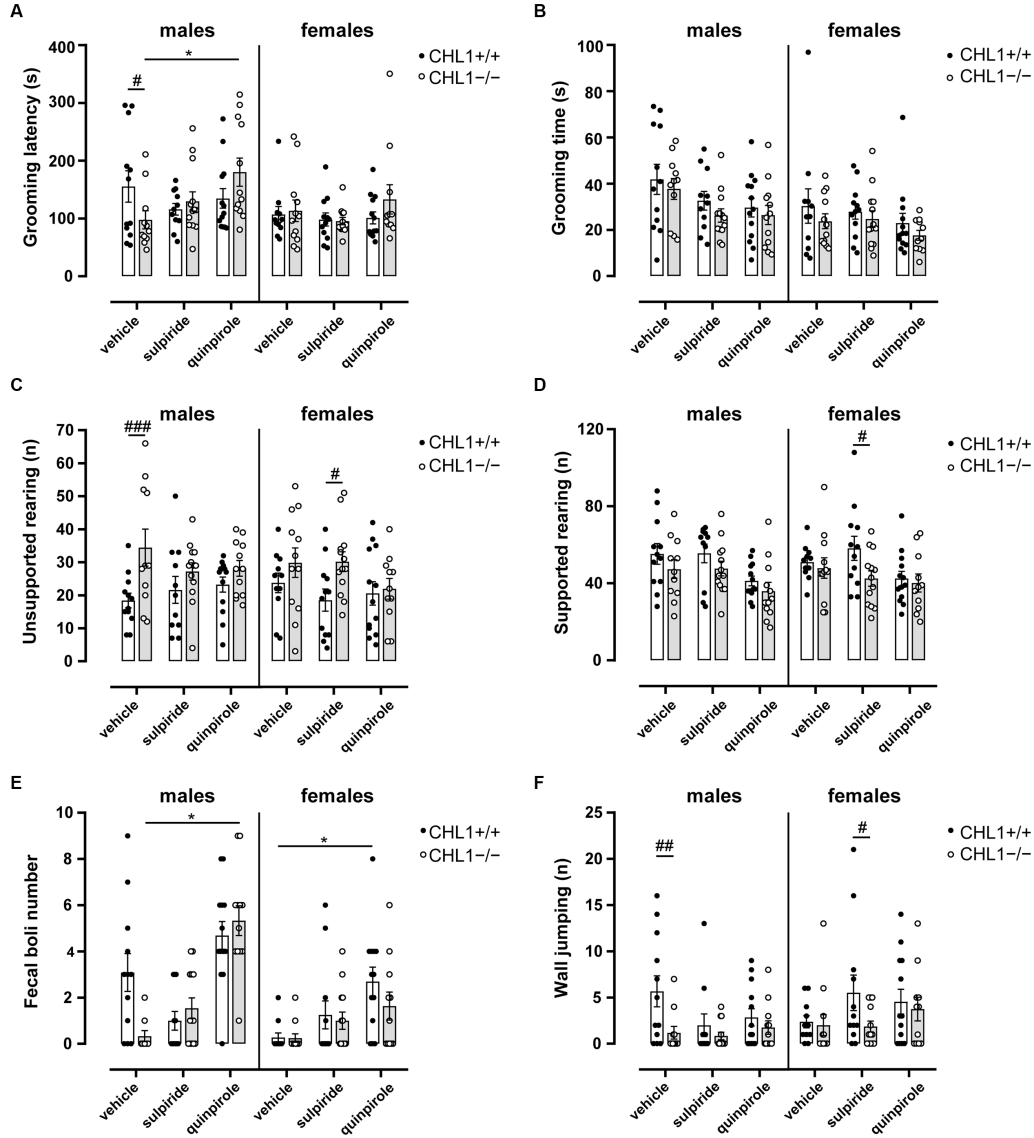
Altered emotional state of male CHL1−/− mice. Three-month-old CHL1+/+ and CHL1−/− males and females were treated with single injections of vehicle, sulpiride (1 mg/kg body weight) or quinpirole (0.02 mg/kg body weight), and activity in the open field was observed over 30 min. Self-grooming latency **(A)**, self-grooming time (**B**; also shown in Figure 4C for sex comparisons), unsupported rearing **(C)** and supported rearing **(D)** were determined during the first 10 min in the open field. Fecal boli deposits **(E)** were determined over 30 min and numbers of jumps on the wall **(F)** were determined during the first 10 min. Values are presented as single values and mean ± SEM (*n* = 11–13 mice per group) and were analyzed with three-way ANOVA (variables: genotype, treatment and sex) followed by the Bonferroni correction *post-hoc* test (*p < 0.05, statistical difference from vehicle-treated correspondent genotype; ^#^*p* < 0.05, ^##^*p* < 0.01, ^###^*p* < 0.001 in case of genotype differences within treatment). White bars: CHL1+/+ mice, grey bars: CHL1−/− mice.

Stress- and novelty-induced exploratory behavior as well as stereotyped behavior of mice can give an indication of the emotional state of the animals. Rearing (vertical activity) is a variant of the search phase in exploratory behavior, when the animal is moving around the environment attempting to contact environmental cues that need to be recognized by the animal to adjust its behavior, and it is an important parameter of the exploratory and motor activity. Particularly during the first minutes of the test, when the reactivity to novelty is highly triggered, rearing can be observed. Rearing on and off wall can also reveal the emotional state of the mice (Sturman et al., 2018). In addition, defecation, which negatively relates to emotionality in rodents, as well as grooming (low stress comfort grooming or stress-evoked grooming) can also alert to levels of stress and anxiety in the mouse (Archer, 1973; Walsh and Cummins, 1976; Kalueff and Tuohimaa, 2004a,b; Ramos, 2008). Vehicle-treated CHL1−/− males exhibited reduced self-grooming latencies and a higher number of unsupported rearings and performed less wall jumpings compared to the vehicle-treated CHL1+/+ males, while there were no differences between vehicle-treated CHL1−/− and CHL1+/+ females and no differences in grooming time between all groups (Figures 4C, 6A–D,F). Vehicle-treated CHL1−/− males also tended to deposit a lower number of fecal boli than CHL1+/+ males, but this difference was not significant (Figure 6E). CHL1−/− and CHL1+/+ males treated with sulpiride did not differ in their emotional state and showed similar self-grooming, rearing and jumping behaviors (Figures 4C, 6A–F). Quinpirole treatment enhanced the latency for self-grooming of CHL1−/− males and led to enhanced deposition of fecal boli, but did not change the self-grooming activity of CHL1+/+ males nor their deposition of fecal boli, grooming time, number of supported and unsupported rearings and wall jumping of males from both genotypes (Figures 4C, 6A–F). CHL1−/− females treated with sulpiride showed more unsupported rearings and less supported rearings compared to sulpiride-treated CHL1+/+ females, but there were no differences between vehicle-treated and sulpiride-treated CHL1−/− females (Figures 6C,D). Quinpirole-treated CHL1+/+ females deposited more fecal boli compared to vehicle-treated CHL1+/+ females, but not to vehicle-treated CHL1−/− females (Figure 6E). Sulpiride-treated CHL1+/+ females jumped more often compared to sulpiride-treated CHL1−/− females, but this parameter was not different between vehicle-treated CHL1+/+ females and sulpiride-treated CHL1+/+ females (Figure 6F). Vehicle-treated CHL1−/− males groomed longer times compared to vehicle-treated CHL1−/− females (Figure 4C). Results suggest that stress-related behavior and emotionality of CHL1+/+ and CHL1−/− males differ, while emotionality of CHL1−/− females is like that of CHL1+/+ females. Interestingly, quinpirole treatment affected stress-related behavior and emotionality of CHL1−/− males, but not of CHL1+/+ males, although quinpirole-induced hypolocomotion was more pronounced in CHL1+/+ males.

### 3.2. Y-maze

Previous studies relate the ablation of CHL1 in mice with impairments of working memory duration, resulting in slower processing speed in the reinforced alternation task (Kolata et al., 2008) and in the intra-trial interval T-maze (Morellini et al., 2007). With a view on the importance of DA and its receptors D1R and D2R in memory formation and cognition, for instance spatial working memory formation (Robbins, 2000; Mehta and Riedel, 2006), we were interested in exploring this relationship further in the context of CHL1 ablation. D1Rs dominate neural responses during stable periods of short-term memory maintenance (requiring attentional focus), while D2Rs play a more specific role during periods of instability such as changing environmental or memory states (requiring attentional disengagement) (Matzel and Sauce, 2023).

#### 3.2.1. Improved working memory of CHL1−/− males and females

**Figure 7:**
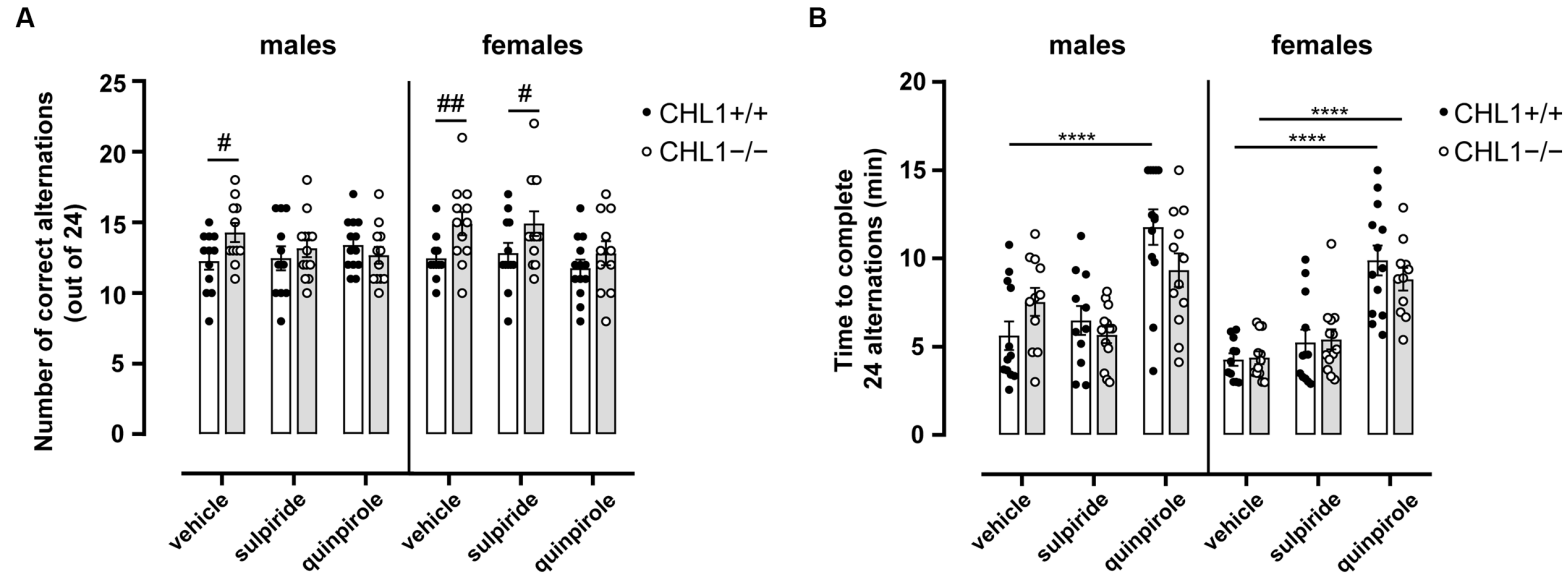
Improved working memory of male and female CHL1−/− mice in the Y-maze. Three-month-old male and female CHL1+/+ and CHL1−/− mice were treated with single injections of vehicle, sulpiride (1 mg/kg body weight) or quinpirole (0.02 mg/kg body weight), and activity in the Y-maze was observed over 15 min. The number of correct alterations **(A)** and time to complete 24 alternations **(B)** were determined. The 15 min values are presented as single values and mean ± SEM (*n* = 11–13 mice per group) and were analyzed with three-way ANOVA (variables: genotype, treatment, and sex) followed by the Bonferroni correction **(A)** and the Brown-Forsythe ANOVA followed by the Games-Howell **(B)** *post-hoc* tests (****p < 0.0001, statistical difference from vehi-cle-treated correspondent genotype; ^#^*p* < 0.05, ^##^*p* < 0.01, difference between genotypes). White bars: CHL1+/+ mice, grey bars: CHL1−/− mice.

To assess the ability of CHL1+/+ and CHL1−/− mice to retain short-term memory, specifically spatial working memory, animals were subjected to the Y-maze after treatment with vehicle, quinpirole or sulpiride. When mice are placed in a three-arm maze, they explore previously unvisited areas due to their innate curiosity for novelty. With intact working memory, animals remember the previously visited arm and tend to enter a less recently visited arm (Lalonde, 2002; Kraeuter et al., 2019). While vehicle-treated CHL1+/+ males and females performed at chance level (50% of correct alternations) (vehicle-treated CHL1+/+ males 51.04 ± 2.52% and vehicle-treated CHL1+/+ females 51.89 ± 1.86% correct alternations), vehicle-treated CHL1−/− males and females performed more correct alterations and above chance level (vehicle-treated CHL1−/− males 59.49 ± 2.81% and vehicle-treated CHL1−/− females 62.15 ± 3.42% correct alternations) (Figure 7A). Sulpiride- and quinpirole-treated CHL1−/− and CHL1+/+ males were similar in numbers of alternations (sulpiride-treated CHL1+/+ males 51.89 ± 3.53%, sulpiride-treated CHL−/− males 54.81 ± 2.53%, quinpirole-treated CHL1+/+ males 57.43 ± 1.96% and quinpirole-treated CHL−/− males 52.78 ± 2.48% correct alternations), but values did not differ from those of vehicle-treated males. Sulpiride-treated CHL1−/− females performed better compared to CHL1+/+ females treated with sulpiride, while quinpirole-treated CHL1−/− and CHL1+/+ females showed the same performance and carried out similar numbers of correct alternations (sulpiride-treated CHL1+/+ females 53.47 ± 3.07%, sulpiride-treated CHL−/− females 62.18 ± 3.64%, quinpirole-treated CHL1+/+ females 49.04 ± 2.50% and quinpirole-treated CHL−/− females 53.41 ± 3.55% correct alternations) (Figure 7A). Interestingly, the time to complete 24 alternations was similar for CHL1+/+ and CHL1−/− males and females and was increased for quinpirole-treated CHL1+/+ males and females and CHL1−/− females, but not for CHL1−/− males (Figure 7B). Results suggest that the better short-term memory performance of CHL1−/− mice was abolished by quinpirole treatment, whereas the performance of CHL1+/+ mice was not altered by quinpirole treatment. We propose that the longer time that quinpirole-treated mice needed to complete 24 alternations is linked to hypolocomotion induced by this drug (Figure 7B, Supplementary Figures S5A,B).

#### 3.2.2. Hypolocomotion of CHL1+/+ but not CHL1−/− mice induced by quinpirole

Next, we analyzed the distance moved by the mice in the Y-maze. As seen in the open field, sulpiride treatment did not alter the distance moved of CHL1+/+ and CHL1−/− males and females. In contrast, quinpirole treatment led to hypolocomotion of CHL1+/+ males and females and reduced locomotion of CHL1−/− females to a lower extent and had almost no effect on CHL1−/− males (Supplementary Figures S5, S6). In the Y-maze, vehicle-treated CHL1−/− females traveled a larger distance than vehicle-treated CHL1−/− males, and sulpiride-treated CHL1+/+ females traveled a larger distance than sulpiride-treated CHL1+/+ males (Supplementary Figures S5B, S6). In contrast to the open field, in the Y-maze CHL1−/− females did not show a delayed hypolocomotion after quinpirole application compared to CHL1+/+ females (Supplementary Figure S5). The effects of quinpirole on locomotion in the Y-maze correspond with those of quinpirole regarding the time needed to perform 24 alternations and the values in the open field where hypolocomotion was seen for males and females of both genotypes.

### 3.3. Novel object test

**Figure 8:**
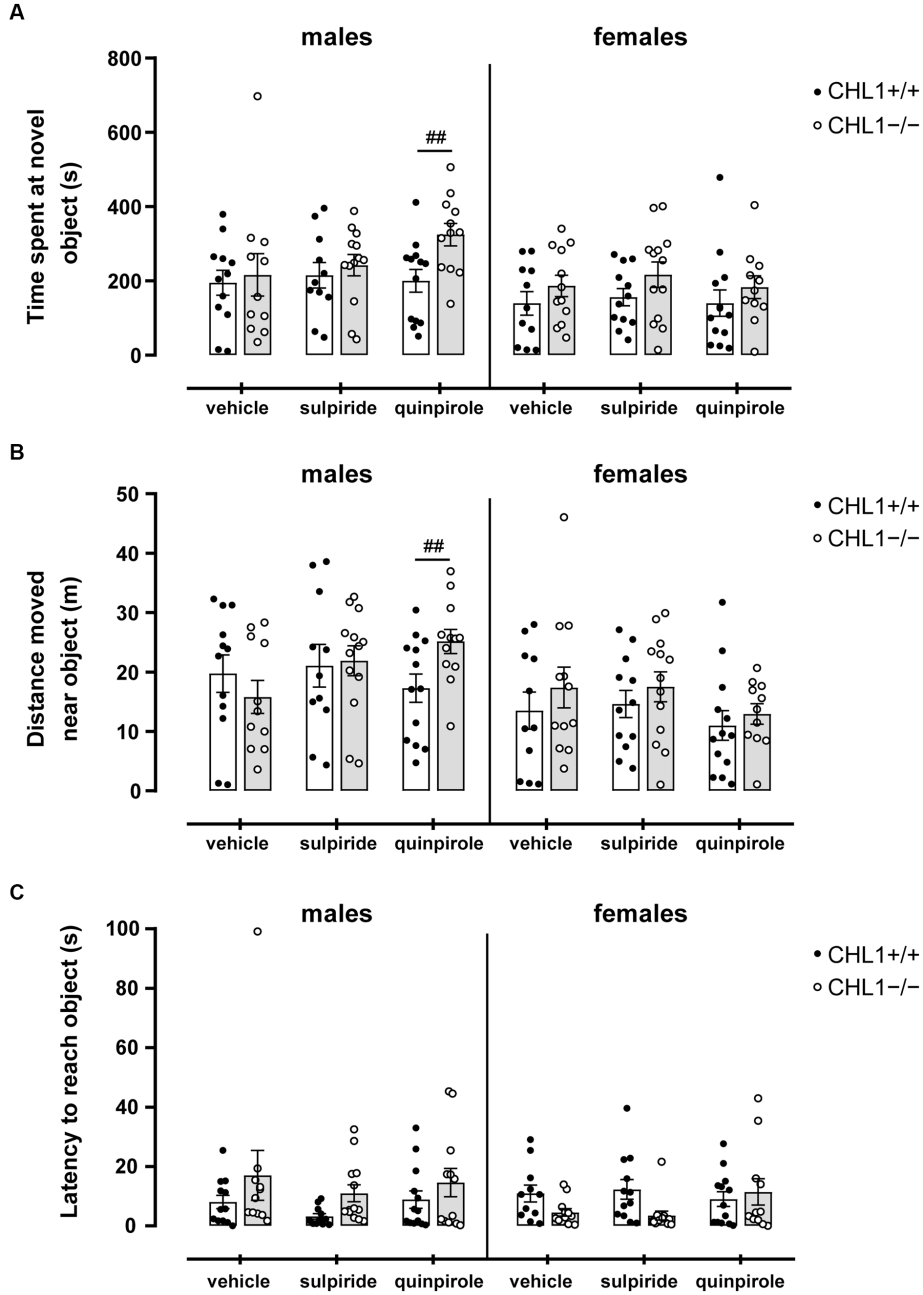
Quinpirole-treated CH1−/− males, but not CHL1−/− females, spent a longer time and move a larger distance near a novel object compared to CHL1+/+ males. Four-month-old male and female CHL1+/+ and CHL1−/− mice were treated with single injections of vehicle, sulpiride (1 mg/kg body weight) or quinpirole (0.02 mg/kg body weight), and activity in the novel object test was recorded for 20 min. Time spent near the novel object **(A)**, distance moved near the novel object **(B)** and latency to reach the object **(C)** were determined. The 20 min values are presented as single values and mean ± SEM (*n* = 11–13 mice per group) and were analyzed with three-way ANOVA (variables: genotype, treatment, and sex) followed by the Bonferroni correction *post-hoc* test (^##^*p* < 0.01, difference between genotypes). White bars: CHL1+/+ mice, grey bars: CHL1−/− mice.

In addition to influencing locomotor activity, D2R availability or pharmacological modulation can directly influence novel stimulus and novelty-seeking behavior (Czoty et al., 2010; Franca et al., 2016). The ablation of CHL1 in mice has also been linked to a mild impairment of novelty-seeking behavior, where an initial hesitation to explore a new object was found for CHL1−/− mice, while no effect on the total time of object exploration was detected (Pratte et al., 2003; Morellini et al., 2007). Thus, we analyzed the behavior of CHL1+/+ and CHL1−/− mice treated with vehicle, quinpirole or sulpiride in the novel object test. After treatment, mice were placed in an arena with a novel object in the center, and the interaction with the novel object was investigated. Vehicle-treated CHL1+/+ and CHL1−/− males and females spent similar times at the novel object, moved the same distance near the novel object and were similarly latent to approach the novel object (Figures 8A–C). Sulpiride and quinpirole treatment did not influence the behavior of CHL1+/+ and CHL1−/− males and females compared to the corresponding vehicle-treated mice of the same genotype (Figures 8A–C). Nonetheless, quinpirole-treated CHL1−/− males spent a longer time at the novel object and moved a larger distance close to the novel object compared to the respective CHL1+/+ males (Figures 8A,B). Also, CHL1−/− males treated with quinpirole spent a longer time near the novel object compared to quinpirole-treated CHL1−/− females, while quinpirole-treated CHL1+/+ mice did not show sex-dependent differences (Supplementary Figure S7). These results indicate an unaltered novelty-seeking behavior of vehicle-treated CHL1−/− mice and suggest that activation of D2R by quinpirole enhanced interest in the novel object in CHL1−/− males but not CHL1−/− females. Of note, blocking of D2R with sulpiride did not change the novelty-seeking behavior of male and female CHL1+/+ and CHL1−/− mice.

We also determined the distance moved and average speed of animals in the novel object test. As seen in the open field and Y-maze, vehicle-treated CHL1−/− males tended to move shorter distances and had a reduced speed compared to vehicle-treated CHL1+/+ males, whereas vehicle-treated females moved a similar distance and at a similar speed (Supplementary Figures S8A,B). Quinpirole induced hypolocomotion of CHL1+/+ males, CHL1+/+ females and CHL1−/− females, but did not affect CHL1−/− males. Quinpirole-treated CHL1−/− males traveled a longer distance compared to the quinpirole-treated CHL1−/− females. In agreement with the findings from the open field test, sulpiride treatment reduced the distance moved and average speed of CHL1−/− females, but did not affect CHL1+/+ females nor CHL1+/+ males and CHL1−/− males (Supplementary Figures S8A,B).

### 3.4. Social interaction

**Figure 9:**
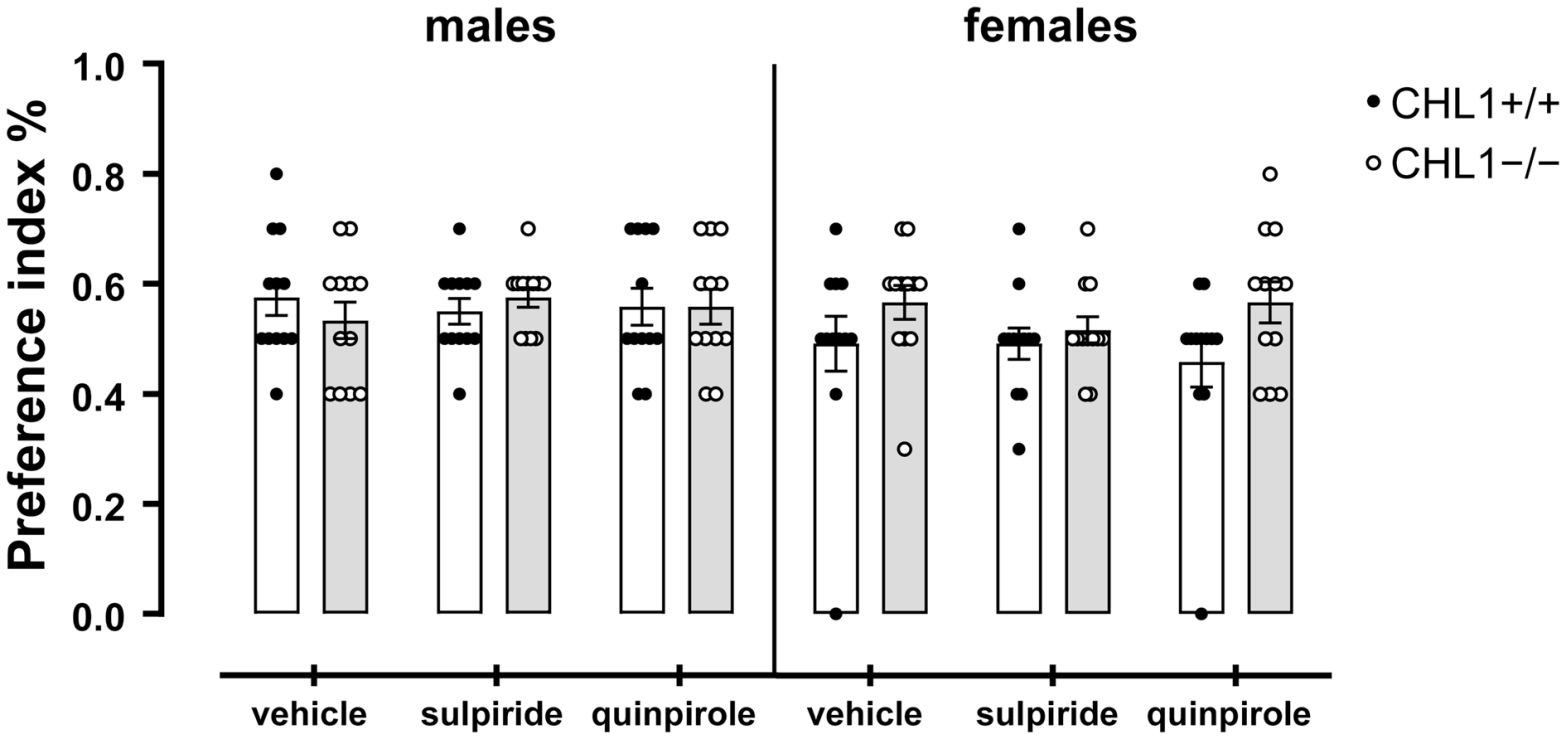
CHL1−/− and CHL1+/+ mice prefer to visit the unfamiliar mouse in the social interaction test. Four-month-old male and female CHL1+/+ and CHL1−/− mice were treated with single injections of vehicle, sulpiride (1 mg/kg body weight) or quinpirole (0.02 mg/kg body weight), and activity in social interaction test was recorded for 20 min. The preference indices were calculated and the 20 min values are presented as single values and mean ± SEM (*n* = 11–13 mice per group). Analysis was carried out for each sex with two-way ANOVA (variables: genotype and treatment) followed by the Bonferroni correction *post-hoc* test. White bars: CHL1+/+ mice, grey bars: CHL1−/− mice.

**Figure 10:**
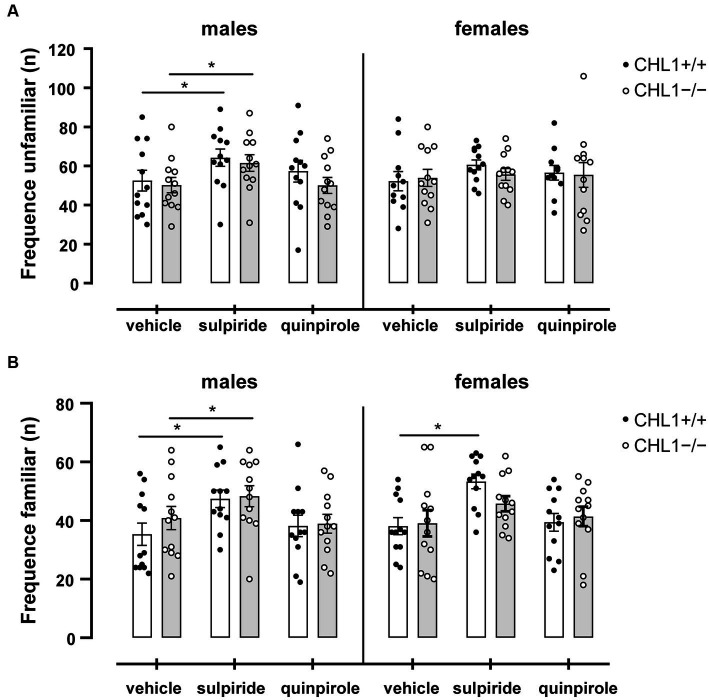
CHL1−/− and CHL1+/+ mice visit the familiar and unfamiliar mouse at a similar frequency. Four-month-old male and female CHL1+/+ and CHL1−/− mice were treated with single injections of vehicle, sulpiride (1 mg/kg body weight) or quinpirole (0.02 mg/kg body weight), and activity in social interaction test was recorded for 20 min, and number of visits at the unfamiliar mouse **(A)** and the familiar mouse **(B)** were counted. Values are presented as mean single values and ± SEM (*n* = 11–13 mice per group) and values from each sex were analyzed with two-way ANOVA (variables: genotype and treatment) followed by the Bonferroni correction *post-hoc* test (*p < 0.05 statistical difference from vehicle-treated correspondent genotype). White bars: CHL1+/+ mice, grey bars: CHL1−/− mice.

Dopamine influences social behavior and social cognition, and this influence can be mediated by D2R (Yamaguchi et al., 2017; Cutando et al., 2022; Mandic-Maravic et al., 2022). Patients with autism spectrum disorder display impaired social interactions and repetitive behavior in addition to cognitive disabilities, anxiety, sleep disturbances, hyperactivity, and motor impairments (see for instance, Lord et al., 2020). Since CHL1 interacts with D2R and regulates its levels at the cell surface (Kotarska et al., 2020), and since CHL1 is also implicated in autism spectrum disorders (Salyakina et al., 2011; Mandic-Maravic et al., 2022), we investigated if treatment of CHL1+/+ and CHL1−/− mice with quinpirole or sulpiride alters their social behavior. CHL1−/− and CHL1+/+ males and females preferred to visit the unfamiliar mouse compared to the familiar mouse in the social interaction test (Figure 9). Treatment with the D2R agonist and antagonist did not change this preference of the CHL1+/+ and CHL1−/− mice (Figure 9). Furthermore, vehicle- and quinpirole-treated males and females of both genotypes visited the familiar and unfamiliar mouse with the same frequency (Figure 10). However, treatment of CHL1+/+ and CHL1−/− males with sulpiride enhanced the number of visits at the familiar and unfamiliar mouse (Figure 10). Interestingly, treatment of CHL1+/+ females with sulpiride enhanced the frequency of visits at the familiar mouse, but did not change the frequency of visits at the unfamiliar mouse. Sulpiride treatment did not change the frequency of visits of the CH1−/− females (Figure 10).

## 4. Discussion

We here examined the interplay of D2R and CHL1 in mice and analyzed if their interaction influences novelty-seeking, exploration, anxiety-related behavior, social interaction and locomotor activity. Interest in novelty, spatial working memory, social interaction and anxiety-related behavior were not different between vehicle- and quinpirole-treated CHL1+/+ and CHL1−/− males and females, while sulpiride treatment enhanced the number of visits at the familiar and unfamiliar mouse by CHL1+/+ males and CHL−/− males but not CHL−/− females. CHL1−/− males and females were delayed in their response to low doses of quinpirole, which was most evident in the open field, where CHL1−/− mice were delayed in hypolocomotion. Interestingly, only CHL1−/− males showed more stress-related behavior after quinpirole treatment and only CHL1−/− females exhibited hypolocomotion and altered anxiety- and stress-related behavior when treated with sulpiride, suggesting that the interaction of CHL1 with D2R modifies exploratory and locomotor activity as well as emotional behavior differently in males and females.

### 4.1. CHL1 and D2R interaction

In our previous studies we observed that the extracellular domain of CHL1 interacts with the first extracellular D2R loop. We propose that this interaction occurs in trans-orientation, since the recombinant extracellular CHL1 domain interacts with D2Rs at the cell surface (Kotarska et al., 2020). CHL1 is present at the cell soma, at dendrites and axons and accumulates presynaptically, while D2R is present pre- and postsynaptically (Leshchyns'ka et al., 2006; Beaulieu and Gainetdinov, 2011; Anzalone et al., 2012). High D2R expression is detectable in the striatum, the nucleus accumbens, and the olfactory tubercle as well as the substantia nigra, ventral tegmental area, hypothalamus, cortical areas, septum, amygdala, and hippocampus (Beaulieu and Gainetdinov, 2011), regions that also express CHL1. Of note, CHL1 reduces quinpirole-triggered internalization of the short D2R, but not the long D2R isoform (Kotarska et al., 2020). Since the short D2R isoform is highly expressed in DAergic neurons pre- and postsynaptically and regulates presynaptic inhibitory feedback control and postsynaptic neurotransmission, it is possible that CHL1 regulates pre- and postsynaptic functions via its interaction with the D2R short isoform. We find it interesting in this context that the dorsal striatum controls motor and cognitive functions, while the ventral striatum regulates motivation, reward, and emotion (Chen et al., 2020). In CHL1−/− mice decreased levels of D2R, enhanced D2 autoreceptor internalization and dysregulation of D2R-dependent DA transporter surface expression and activity in the dorsal striatum could lead to altered presynaptic signaling and decreased levels of phosphorylated tyrosine hydroxylase, leading to enhanced feedback inhibition and reduced motor activity in CHL1−/− mice. Since CHL1 stabilizes the short D2R isoform at the cell surface upon activation with quinpirole (Kotarska et al., 2020), we would like to suggest that lack of CHL1 leads to altered D2R signaling or ligand binding affinities that might change behavior. The findings that CHL1 co-localizes with D2R in DA- and cyclic-AMP-regulated phosphoprotein of molecular weight 32,000 (DARPP-32)-positive striatal cells and that lower D2R and phosphorylated tyrosine hydroxylase levels are seen in the dorsal striatum of CHL1−/− mice, whereas in the ventral striatum levels of phosphorylated DARPP-32 are reduced compared to levels in CHL+/+ mice (Kotarska et al., 2020), support this speculation. In the following sections we attempt an interpretation how the interplay of D2R and CHL1 might influence behavior in sex-specific and sex-unspecific manner.

### 4.2. Sex differences in DAergic signaling and DA-related behaviors

Sex differences in DAergic signaling and DA-related behaviors as well as in several neuropsychiatric disorders have been acknowledged in numerous studies over many years (Williams et al., 2021, and references therein). Animal studies showed that the DA systems differ between males and females and display differences in DA release and DA receptor expression in adulthood and during development. DA receptor agonists and antagonists trigger sex-specific functional responses and sex-specific differences in behavior in adulthood during decision-making, learning, anxiety and reward (Williams et al., 2021, and references therein). Many neuropsychiatric disorders are characterized by a dysfunction of the DAergic system in reward-related brain regions (Williams et al., 2021, and references therein). Apart from regulation of the DAergic system by ovarian hormones in females, many sex differences are independent of the ovarian cycle and are due to differences in DAergic system function and structural (re-)organization, showing different distribution of DAergic neurons in the midbrain, leading to increased DA receptor activity, increased release of DA from synaptic terminals in the striatum and increased basal DA levels (Zachry et al., 2021, and references therein). In the striatum, DA release from terminals is maintained in the adult and controlled by regulatory proteins, including D2 autoreceptors and DA transporters (Zachry et al., 2021, and references therein). These regulatory proteins participate in hormonal and non-hormonal homosynaptic and heterosynaptic regulation of DA release and trigger or inhibit DA release and modulate the timing and magnitude of the release (Zachry et al., 2021, and references therein). Sex-dependent expression and activity of these regulatory proteins may regulate DA signaling and DA-dependent behaviors differently in males and females. The DA transporter contributes to the clearance of DA from synaptic and extra-synaptic spaces and hence plays an important role in the timing and duration of DA-triggered events (Walker et al., 2006). It has been reported that D2R regulates the surface expression and activity of the DA transporter and thus, the DA clearance in a sex-dependent manner (Stewart et al., 2021). In males, D2R-dependent regulation of the DA transporter predominantly takes place in the dorsal striatum containing projections of DAergic neurons from the substantia nigra, whereas this regulation does not occur in the ventral striatum. In females, regulation of the DA transporter by D2R is detectable in the ventral striatum containing projections of DAergic neurons from the ventral tegmental area, whereas this regulation was not evident in the dorsal striatum (Stewart et al., 2021).

Like CHL1−/− males, mice lacking the long D2R isoform were reduced in locomotion compared to mice expressing both D2R isoforms (Wang et al., 2000). These results suggest that the interplay between CHL1 and the long D2R isoform is also crucial for regulating DA-driven striatum-dependent locomotion. In CHL1−/− females, absence of CHL1 could inhibit internalization of postsynaptic D2Rs in the ventral striatum and may alter the conformation of postsynaptic D2R, leading to an altered sensitivity to sodium ions, which decreases the affinity of DA and DA agonists and enhances the affinity for several antagonists (Michino et al., 2015). Interestingly, the affinity of wild-type D2R for sulpiride increases ~23-fold in the presence of sodium, while the affinity of sulpiride to D2R mutants was not altered (Wilson et al., 2001; Michino et al., 2015). It is likely that in CHL1−/− females the postsynaptic D2Rs in the ventral striatum could be more sensitive to sulpiride. Sulpiride can indeed function as inverse agonist that binds to the same receptor as an agonist, but induces a response opposite to that of an agonist (Hall and Strange, 1997; Zhang et al., 2014). Thus, we propose that sulpiride triggers postsynaptic responses opposite to DA because of its increased affinity for D2R, thereby reducing postsynaptic activity accompanied by reduced levels of phosphorylated DARPP-32 that lead to changes in motivation-driven locomotion and in emotionality. Of note, sulpiride treatment reduced locomotor activity in the open field and Y-maze, reduced numbers of supported rearing and reduced wall jumping in CHL1−/− females relative to CHL1+/+ females, whereas there was no difference between sulpiride-treated CHL1+/+ and CHL1−/− males.

### 4.3. Locomotor activity

Beside sex-specific differences in behavior, we also observed a sex-independent difference of locomotor activity at early stages after application of low quinpirole doses which mainly act on presynaptic D2 autoreceptors (Boschen et al., 2011, 2015). Application of a low dose of the DA agonist quinpirole stimulates selectively D2 autoreceptors and leads to an early suppression of locomotor activity (Mogenson and Wu, 1991a,b; Frantz and Van Hartesveldt, 1995; Horvitz et al., 2001; Schindler and Carmona, 2002; Anzalone et al., 2012). Mice treated with a low dose of quinpirole were reduced in exploratory behavior and enhanced in immobility but not changed in flight reaction, while mice treated with higher doses of quinpirole were altered in flight behavior (Gao and Cutler, 1993). In addition, quinpirole treatment induced hypolocomotion of wild-type mice and mice lacking the long D2R isoform at lower concentrations compared to mice lacking the short D2R isoform (Radl et al., 2018). In our current study, CHL1+/+ males and females showed a decrease in locomotion within the first 6–7 min in the open field and Y-maze after injection of a low concentration of quinpirole, indicating that a low dose of quinpirole inhibits locomotion by stimulating presynaptic feedback inhibition via D2 autoreceptor signaling and internalization. Yet, in CHL1−/− males and females quinpirole treatment did not lead to this early suppression of locomotion. It is interesting in this context that CHL1 inhibited quinpirole-triggered internalization of the short D2R isoform but not the long isoform and led to higher cell surface expression of the short D2R isoform (Kotarska et al., 2020), indicating that in CHL1−/− mice less D2R is expressed at the cell surface after stimulation, that the presynaptic D2 autoreceptor did not respond to quinpirole in an early phase, that it displays a retarded response or that it is reduced in ligand affinity. Changes in affinity states of the D2R isoforms could contribute to the delayed reaction of CHL1−/− mice to quinpirole and could lead to constitutive activation of presynaptic D2Rs in the dorsal striatum and postsynaptic D2Rs in the ventral striatum.

Sulpiride treatment led to reduced locomotion of CHL1−/− females in the open field during the first 10 min of the test, while CHL1+/+ females and CHL1−/− and CHL1+/+ males showed no alterations compared to vehicle-treated mice. In a study using young male and female wild-type rats, sulpiride injection alone did not alter locomotion, but it antagonized the biphasic locomotor effect of quinpirole (Frantz and Van Hartesveldt, 1995). Furthermore, male wild-type mice did not exhibit altered locomotion after sulpiride treatment, but showed facilitated extinction of conditioned fear (Ponnusamy et al., 2023). These findings suggest that, when applied alone, low doses of sulpiride do not induce opposite effects of quinpirole on D2R and strengthen the notion that D2Rs might function differently in CHL1−/− females compared to males.

### 4.4. Stress-related and social behavior

When evaluating stress-/anxiety-related and social behaviors, sulpiride treatment did not influence stress-related behavior, such as grooming, fecal boli deposition, distance moved in the center or distance to the wall. Yet, sulpiride treatment influenced social behavior in a sex-dependent manner: enhanced numbers of visits at the familiar and unfamiliar mouse were observed for CHL1+/+ and CHL1−/− males, while only enhanced numbers of visits at the familiar mouse were observed for CHL1+/+ females. In agreement with our findings, increased social interaction and unchanged stereotyped behavior of sulpiride-treated rats had been described (Sams-Dodd, 1998). Of note, male and female rats treated with 10 mg/kg sulpiride did not show changes in rearing or grooming behavior, but a higher sulpiride dose reduced these behaviors (Díaz-Véliz et al., 1999). As low doses of sulpiride specifically block presynaptic D2R (Kuroki et al., 1999; Ohmann et al., 2020), one may speculate that the absence of CHL1 in females prevents sulpiride’s impact on presynaptic D2Rs and promotes the blockade of postsynaptic D2Rs, leading to no sulpiride-induced alterations of social behavior in CHL1−/− females. Furthermore, social interactions were not altered by quinpirole treatment. In contrast, quinpirole treatment altered certain behaviors related to stress in a sex-dependent manner: CHL1−/− males showed an enhanced grooming latency and CHL1−/− males and CHL1+/+ females showed enhanced fecal boli depositions when treated with quinpirole, while CHL1+/+ females, CHL1−/− females and CHL1+/+ males displayed a reduced distance moved in the center. Distance to the wall and time in the center did not differ between genotypes and treatments, suggesting that quinpirole but not sulpiride changed stress-related behavior especially of males. Similar results had been described before: low quinpirole doses of 0.05 and 0.1 mg/kg did not change rearing, grooming or jumping behavior of male mice, while treatment with 1 mg/kg increased rearing and jumping (Luque-Rojas et al., 2013). When mice were tested during the light phase and treated with 0.5 mg/kg quinpirole time in the center was not changed but numbers of rearings were reduced (Moraes et al., 2021). These observations indicate that sulpiride at low concentrations affects mainly social behavior, while stress-related behavior is more affected by quinpirole.

### 4.5. CHL1 and D2R in psychiatric diseases

Dorsoventral parcellation of the striatum is crucial for the expression of sex-specific behavior and for mediating many psychiatric diseases, such as Huntington’s disease, Parkinson’s disease, addiction, depression, anxiety, schizophrenia, attention deficit hyperactivity disorder and autism spectrum disorder. The sex-specific behavioral differences in CHL1−/− mice and CHL1’s implication in schizophrenia and autism spectrum disorders (Sakurai et al., 2002; Frints et al., 2003; Chen et al., 2005; Tam et al., 2010; Salyakina et al., 2011; Shoukier et al., 2013) suggests that CHL1 participates in sex-dependent regulation of DA-dependent pathways in the dorsal and ventral striatum.

### 4.6. Limitations of the study

Systemic administration of quinpirole can cause centrally mediated changes in blood pressure which are associated with changes in plasma levels of noradrenaline and adrenaline (Van den Buuse, 1992, 1995, 1997), which could alter emotionality and activity of mice. These effects of quinpirole are dose-dependent and concentrations below 0.1 mg/kg, as we used here, did not enhance blood pressure (Van den Buuse et al., 1998). While continuous systemic application of quinpirole affected also D1R functions, intermittent quinpirole application did not affect this receptor (Engber et al., 1993). Thus, although we cannot rule out that effects of quinpirole are not only mediated by D2R, it is likely that the intermittent one-time injections of 0.02 mg/kg quinpirole affected mostly D2Rs. D2Rs are found at a high density in the striatum, nucleus accumbens, and olfactory tubercle, and D2 autoreceptors are located on the soma and dendrites of midbrain DAergic neurons in the ventral tegmental area and substantia nigra pars compacta as well as on their axon terminals in projection areas. Low levels of D2Rs are expressed in the hippocampus, amygdala, hypothalamus and cortical regions. Activation of D2 autoreceptors on midbrain DAergic neurons reduced locomotion in the current study, and this mode of action was confirmed using conditional knock-out mice in which D2Rs were deleted only from DAergic neurons (Anzalone et al., 2012). These findings suggest that indeed the hypolocomotion observed after quinpirole treatment and the differences in response of CHL1−/− and CHL1+/+ mice are mediated by D2Rs on DAergic neurons. CHL1−/− mice used in the current study exhibit increased numbers of excitatory spine synapses (Mohan et al., 2019), defects in tyrosine hydroxylase-positive axonal projections during embryonic development (Alsanie et al., 2017) and abnormally high numbers of parvalbumin-expressing hippocampal interneurons at juvenile age (Schmalbach et al., 2015). These changes could influence the behavior of adult mice and affect the response to stimulation of D2Rs. Specific deletion of CHL1 from DAergic neurons in adulthood could more specifically advance insights into the influence of CHL1 on D2Rs.

## 5. Conclusion

In the open field, Y-maze and novel object test CHL1−/− male and female mice are delayed in responding to low doses of the D2R agonist quinpirole, whereas anxiety, working memory and novelty-seeking behavior are similar to that of CHL1+/+ males and females. CHL1−/− males exhibit more stress-related behavior after quinpirole treatment and CHL1−/− females respond with hypolocomotion and altered emotionality when treated with the D2R antagonist sulpiride. These results indicate that differences between males and females in the interaction of CHL1 with D2R influence exploratory and locomotor activities and emotional behavior. We hypothesize that CHL1 regulates D2R-dependent functions, thereby affecting behavior in a sex-dependent as well as sex-independent manner.

## Data availability statement

The raw data supporting the conclusions of this article will be made available by the authors, without undue reservation.

## Ethics statement

The animal studies were approved by Behörde für Justiz und Verbraucherschutz of the State of Hamburg. The studies were conducted in accordance with the local legislation and institutional requirements. Written informed consent was obtained from the owners for the participation of their animals in this study.

## Author contributions

LF: Data curation, Formal analysis, Investigation, Methodology, Validation, Writing – review & editing. RK: Conceptualization, Supervision, Writing – review & editing. LC: Data curation, Formal analysis, Writing – review & editing. SF: Supervision, Writing – review & editing. MK: Resources, Writing – review & editing. GL: Supervision, Validation, Visualization, Writing – original draft, Writing – review & editing. MS: Funding acquisition, Resources, Supervision, Writing – review & editing.
