# Supplementary figures and images for: CHL1 depletion affects dopamine receptor D2-dependent modulation of mouse behavior
Source: Front Behav Neurosci. 2023 Nov 9;17:1288509. doi: 10.3389/fnbeh.2023.1288509 (PMC10665519; doi:10.3389/fnbeh.2023.1288509)

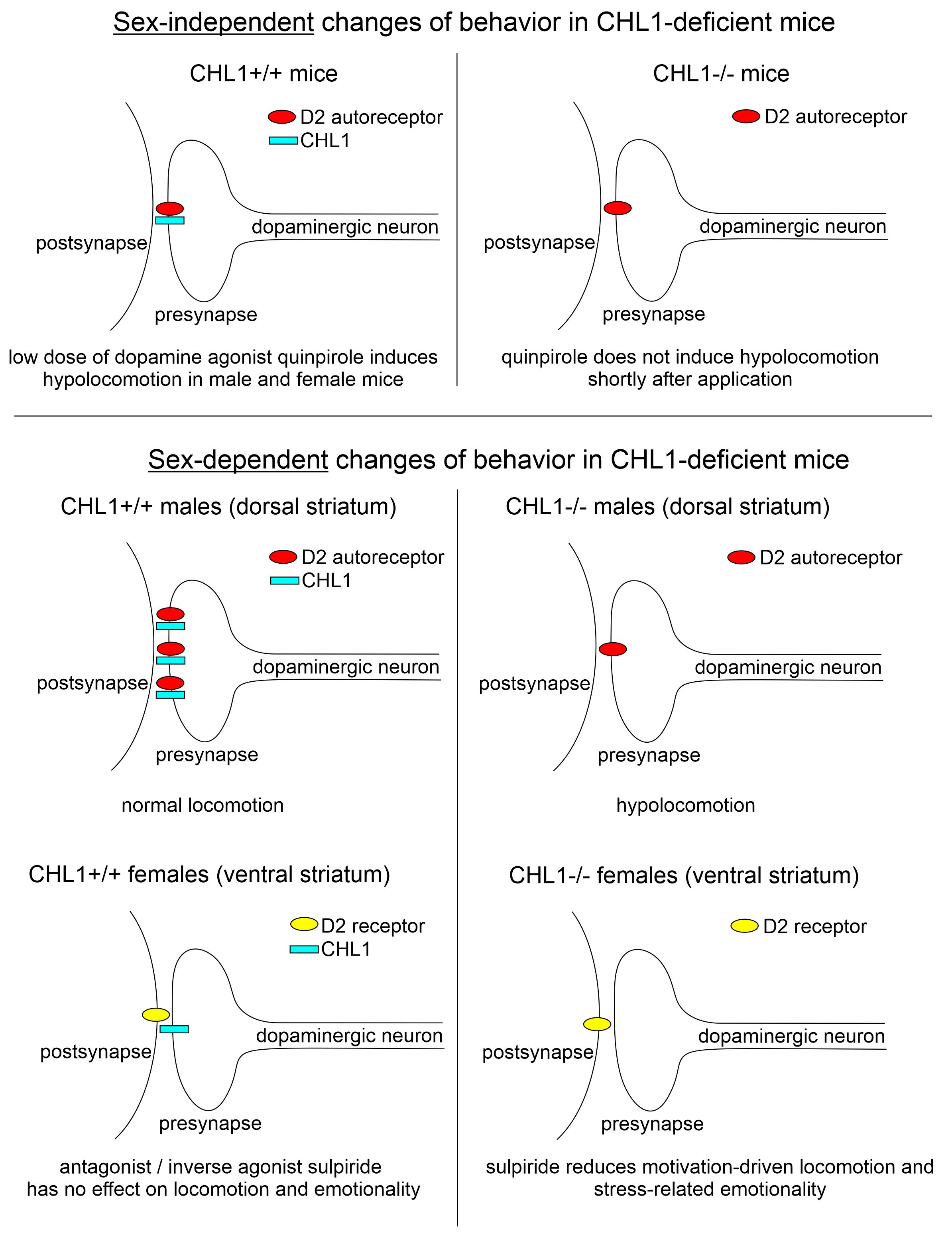

Supplement: Supplementary file 1 [file Image_1.JPEG]
