# Supplementary material for: CHL1 depletion affects dopamine receptor D2-dependent modulation of mouse behavior
Source: Front Behav Neurosci. 2023 Nov 9;17:1288509. doi: 10.3389/fnbeh.2023.1288509 (PMC10665519; doi:10.3389/fnbeh.2023.1288509)
Supplement: Supplementary file 2 [file Data_Sheet_1.pdf]

Supplementary Material for “CHL1 depletion affects dopamine receptor D2-dependent modulation of mouse behavior” by Fernandes, Kleene, Congiu, Freitag, Kneussel, Schachner and Loers

1 Supplementary Methods

CHL1 was shown to interact with both D2R isoforms and to reduce the internalization of the short D2R isoform (Kotarska et al., 2020). The short D2R isoform has a higher affinity for DA and more effectively inhibits adenylyl cyclase than the long isoform. Both D2R isoforms can serve as autoreceptors and activation of these receptors decreases excitability of DA neurons and release of DA leading to a reduction in locomotion (Ford, 2014). On the basis of these findings we hypothesized that CHL1−/− mice might contain fewer functional D2 autoreceptors at the cell surface and display reduced responses after activation of D2Rs. To target especially presynaptic D2 autoreceptors low doses of the D2R agonist quinpirole and antagonist sulpiride were used (Boschen et al., 2015; Boschen et al., 2011; Eilam and Szechtman, 1989; Van Hartesveldt et al., 1994) and D2R-dependent behaviors were investigated. Quinpirole and sulpiride were chosen because of their significantly higher affinity towards D2Rs than D3Rs (Vallone et al., 2000). Sulpiride was chosen as D2R antagonist since it can antagonize the biphasic effect of quinpirole on locomotion (Frantz and van Hartsfeldt, 1995) and opposite effects of quinpirole and sulpiride on D2Rs could therefore be expected.

1.1 Behavior

Since environmental factors such as sound, light, handling and injection can profoundly impact behavior, we tried to minimize these influences. 1. Two-month-old mice were housed in groups of three to four mice consisting of one heterozygous and at least one CHL1+/+ and one CHL1−/− littermate. To control for litter effects, no more than two males or females of the same genotype per litter were used. We housed males and females together in a vivarium with an inverted light-dark cycle. 2. We treated all groups under the same conditions and tests started and ended at least 2 h after light offset and 3 h before light onset. 3. We used consistent light intensity: dim red light with 5 lux, which corresponds to the light intensity in a moonlit environment, in the vivarium during the dark cycle, in the experimental room, the mazes for social interaction and novel object test and 10 or 50 lux for an anxiogenic environment in the center of the Y-maze and open field, respectively. 4. We used a quiet testing room and before the experiments the test animals were transported singly to the experimental room next to the vivarium which was illuminated by dim red light (5 lux) and left for 30 min for habituation. 5. The same equipment was used for all mice and cleaned thoroughly between mice with soap and water and then with 30% ethanol to remove odors of animals that had been in the arena. 6. Mice were accustomed to an inverted light-dark-cycle (light off at 7:00 am) for two weeks and then to the experimenter by 1 h handling and daily body weight monitoring once per day for two weeks.

Sex differences in DA systems were mostly seen to be independent of the ovarian cycle, but due to functional differences in the DAergic system and its structural (re-)organization (Zachry et al., 2021). Previous studies also showed that behavior of females in the open field, tail suspension test, elevated plus maze, novel object, rotarod and hole board were not impacted by the estrus cycle (Chari et al., 2020; Meziane et al., 2007). Thus, we did not determine the estrus cycle, but allowed synchronization of the estrus cycle via the Whitten effect, by housing in groups and home cages were placed next to each other (McClintock, 1978).

Testing began with manual restraint of the mouse using the scruffing technique and a single intraperitoneal injection of 300 µl vehicle solution (sterile filtered 0.9% NaCl, 0.5% dimethyl sulfoxide in water), sulpiride solution (1 mg/kg body weight in vehicle solution), or quinpirole solution (0.02 mg/kg body weight in vehicle solution) per 30 g of body weight using a sterile 27 G needle. The short D2R isoform shows a higher binding affinity for DA and benzamides like sulpiride than the long D2R isoform (Castro and Strange, 1993). The D2R agonist quinpirole has a biphasic action: low doses of quinpirole, as we use here, are suggested to stimulate selectively DA presynaptic autoreceptors and to inhibit locomotor activity. In contrast, high doses of quinpirole initially suppress and later induce locomotion presumably due to stimulation of postsynaptic receptors (Eilam and Szechtman, 1989; Van Hartesveldt et al., 1994). Sulpiride, when administered at lower doses (1 mg/kg body weight), acts primarily at presynaptic D2R (Boschen et al., 2015; Boschen et al., 2011) and is 100-fold more selective for D2R compared to D3R (Vallone et al., 2000). In order to specifically target presynaptic D2R, lower doses of D2R-specific drugs were used. To identify the early onset of presynaptic effects of quinpirole behavioral observations were started 2 min after the injection of the solutions (Anzalone et al., 2012; Eilam and Szechtman, 1989; Frantz and Van Hartesveldt, 1995; Lane et al., 2012; Usiello et al., 2000). Care was taken to minimize pain or discomfort for the animals. Tracks representing the position of the mice were created and analyzed with EthoVision XT (Noldus, Wageningen, The Netherlands; https://www.noldus.com/ethovision; RRID:SCR_000441) (Freitag et al., 2003). Manual scoring of grooming and jumping behavior was performed by a trained experimenter blinded to the genotype and treatment of the mice using The Observer software (Noldus). Numbers of mice used per group are indicated in the figure legends. The timeline of the experiments is presented in Figure 1. Between tests, mice were allowed to recover for 7 days before the next test was started with injection of a single dose of the solutions followed after 2 min by behavioral evaluation in the next test. All experiments were performed with the same batch of mice.

1.2 Open field

The open field (OF) paradigm is a widely used behavioral test used to assess motor activity, exploratory behavior, and anxiety-like or stress-related behavior (Prut and Belzung, 2003; Seibenhener and Wooten, 2015). The test was performed in a square open-field arena (50 × 50 × 50 cm) illuminated by 50 lux. Two min after the injection, the mouse was placed in one corner of the arena. The trial started 2 sec after the first movement of the mouse was detected in the arena and activity of the mouse was recorded for 30 min with a sample rate of 12.5 frames per sec. Parameters were evaluated either across the entire duration of the test or in specific time intervals, including distance moved, time spent in the center of the arena, time moving, average speed, and average distance from wall. In addition, during the first 10 min of the test, frequency, duration and latency of

stereotyped behaviors, including supported and unsupported rearing, self-grooming, and jumping were determined. The self-grooming latency was taken as the amount of time between the first movement in the open field and the start of the self-grooming behavior. Supported rearing was defined as vertical exploration in which the mouse stands on its hind legs with one or two front paws touching the wall, while unsupported rearing refers to the same posture without wall support. Deposition of fecal boli was determined after 30 min in the arena. The anxiety and stress states strongly influence cognitive function of mice, their general behavior and their social interactions, thus this emotional state was evaluated using the parameters time in the center of the open field, on and off wall rearing, grooming, jumping and fecal boli deposition.

1.3 Spontaneous alternation in the Y-maze

To assess short-term memory retention, particularly spatial working memory, spontaneous alternation in a free-trial Y-maze was tested. In a three-arm maze, an intact working memory allows mice to recall which arm was previously visited, resulting in a preference for entering an arm that was less recently visited (Kraeuter et al., 2019; Lalonde, 2002). The Y-maze comprised three identical opaque arms orientated at 120° angles, measuring 30 × 7 cm each, and was illuminated by 10 lux. The test mouse was placed at the end of an arm of the Y-maze 2 min after receiving the injection. The trial began when the mouse reached the center of the maze and the spontaneous movement of the mouse was recorded for 15 min at a sample rate of 12.5 frames per sec. Mice were kept in the maze until they performed 24 correct alternations. For analysis, number of correct alternations, percentage of correct alternations and time to complete 24 correct alternations were obtained. In addition, the distance moved and time moving were analyzed to investigate the locomotion of the animals.

1.4 Novel object test

To target the novelty-seeking behavior triggered by a new stimulus, for which there was no pre-existing recognition memory, the novel object test was used (Akiti et al., 2022; Barto et al., 2013). The test was performed in a square open-field arena (50 × 50 × 50 cm) illuminated by 5 lux. After habituation to the empty arena for 5 min, the mouse was taken back to the home cage and injected with the respective solution. The novel object was inserted in the middle of the arena and 2 min after the injection, the mouse was placed in one corner of the previous arena. The trial started 2 sec after the first movement of the mouse was detected in the arena and was recorded for 20 min. An 8 cm radius was set around the object, defining the object zone. To evaluate the mouse's direct interaction with the object, its nose movement (nose-point) was tracked within the object area. The nose-point movement within the object zone was analyzed to determine distance moved, time spent in the area and time moving. The center-point movement in the whole arena (excluding the object area) was also analyzed to study locomotion. The latency to reach the object was taken as the amount of time between the first movement of the mouse and the time to reach the novel object.

1.5 Social interaction

The motivation to investigate a social stimulus was analyzed by giving the experimental mouse the choice to investigate an unfamiliar mouse or a familiar sex-matched mouse (Freitag et al., 2003). The arena used for the open field test (50 × 50 × 50 cm) illuminated with 5 lux was divided into two identical compartments by a 40 cm high wall with an opening in the middle allowing the mouse access to both compartments. A cylinder with a metal grid mesh allowing olfactory exploration between mice was located in one corner of each compartment containing either a familiar or an

unfamiliar mouse. Familiar mice were recruited from heterozygous siblings living in the same cage as the experimental mice. Unfamiliar mice were heterozygous mice from different cages as the experimental mouse and were not used as subjects in the behavior tests. First, the familiar and unfamiliar mice were placed under the cylinders and then the subject mouse was placed in the arena and left free to move between compartments for 20 min. The times spent in the proximity of the familiar and unfamiliar mouse were used to calculate the preference index and the frequencies to visit the familiar and unfamiliar mouse were determined.

1.6 Statistics

The IBM SPSS Statistics 25 software (IBM, IL, Chicago, USA) and GraphPad Prism 8 software (GraphPad Software, Boston, MA, USA) were used to conduct the statistical analyses. First, the Tukey's fences test was applied to identify outliers, which were included in the analysis and shown in the figures, since significances did not change when the outliers were removed. Next, the assumption of normal distribution was tested using the Shapiro-Wilk test. Values in graphs are presented as mean ± standard error of the mean. The statistical tests used to analyze the data are indicated in the figure legends and their results are presented in the supplementary tables Table S1 and S2. The threshold value for acceptance of differences between group mean values was 5%. Three-way ANOVA was employed when data was normal distributed, and genotype (CHL1+/+ and CHL1−/−), treatment (vehicle, sulpiride and quinpirole) and sex (male and female) were considered between-subjects factors. For pairwise comparisons, the Bonferroni correction post-hoc test was performed when statistically significant differences were identified in the ANOVA test. In cases where data did not follow a normal distribution and failed Levene's test for homogeneity of variance, the Brown-Forsythe ANOVA test was used and the Games-Howell post-hoc test was used for multiple comparisons.

2 Supplementary Figures and Tables

2.1 Supplementary Figures

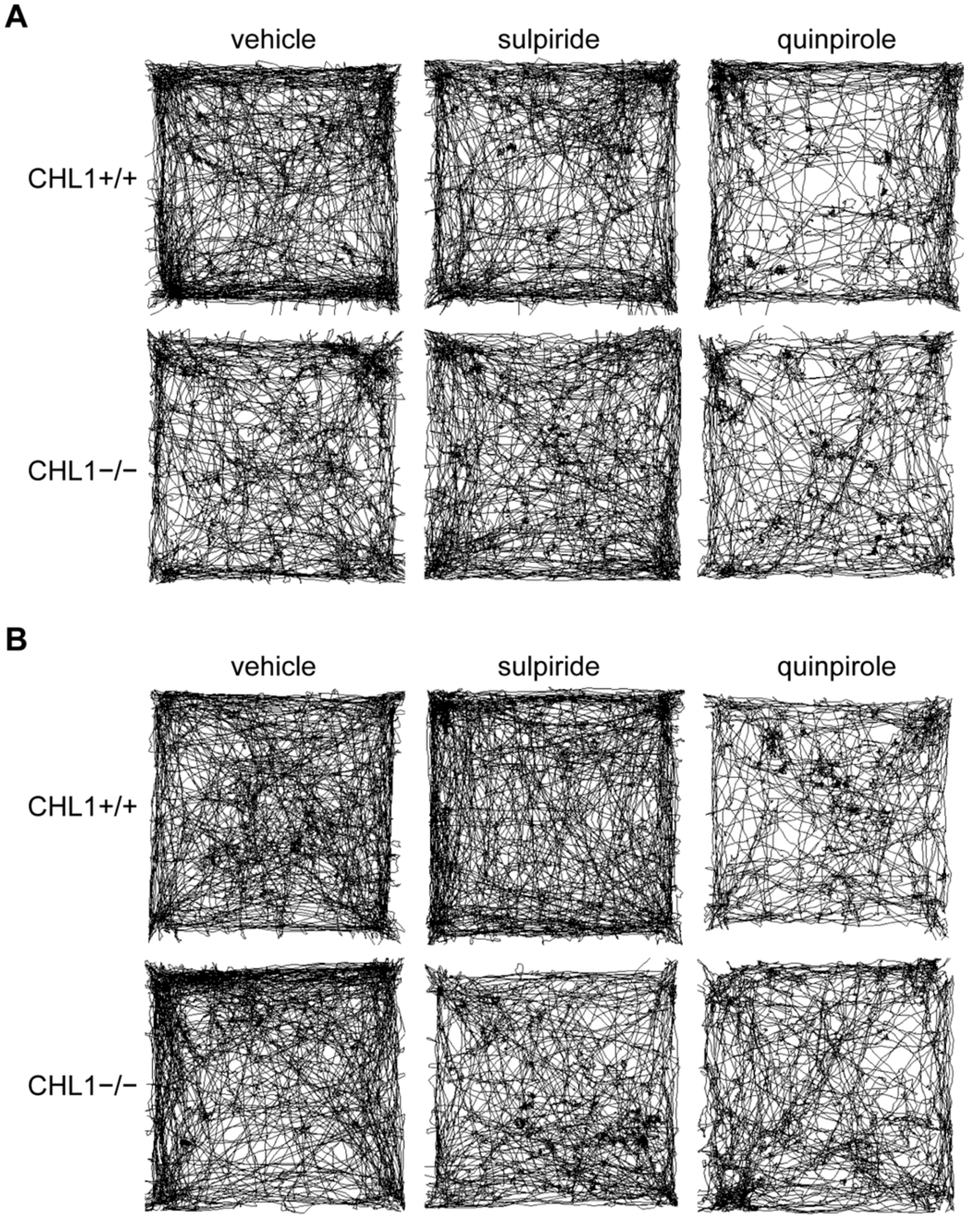

Supplementary Figure S1. Tracks of CHL1+/+ and CHL1−/− males and females in the open field. Locomotor activity of three-month-old vehicle-treated, sulpiride-treated and quinpirole-treated CHL1+/+ and CHL1−/− males (A) and females (B) in the open field was recorded for 30 min and representative tracks of moving mice are shown.

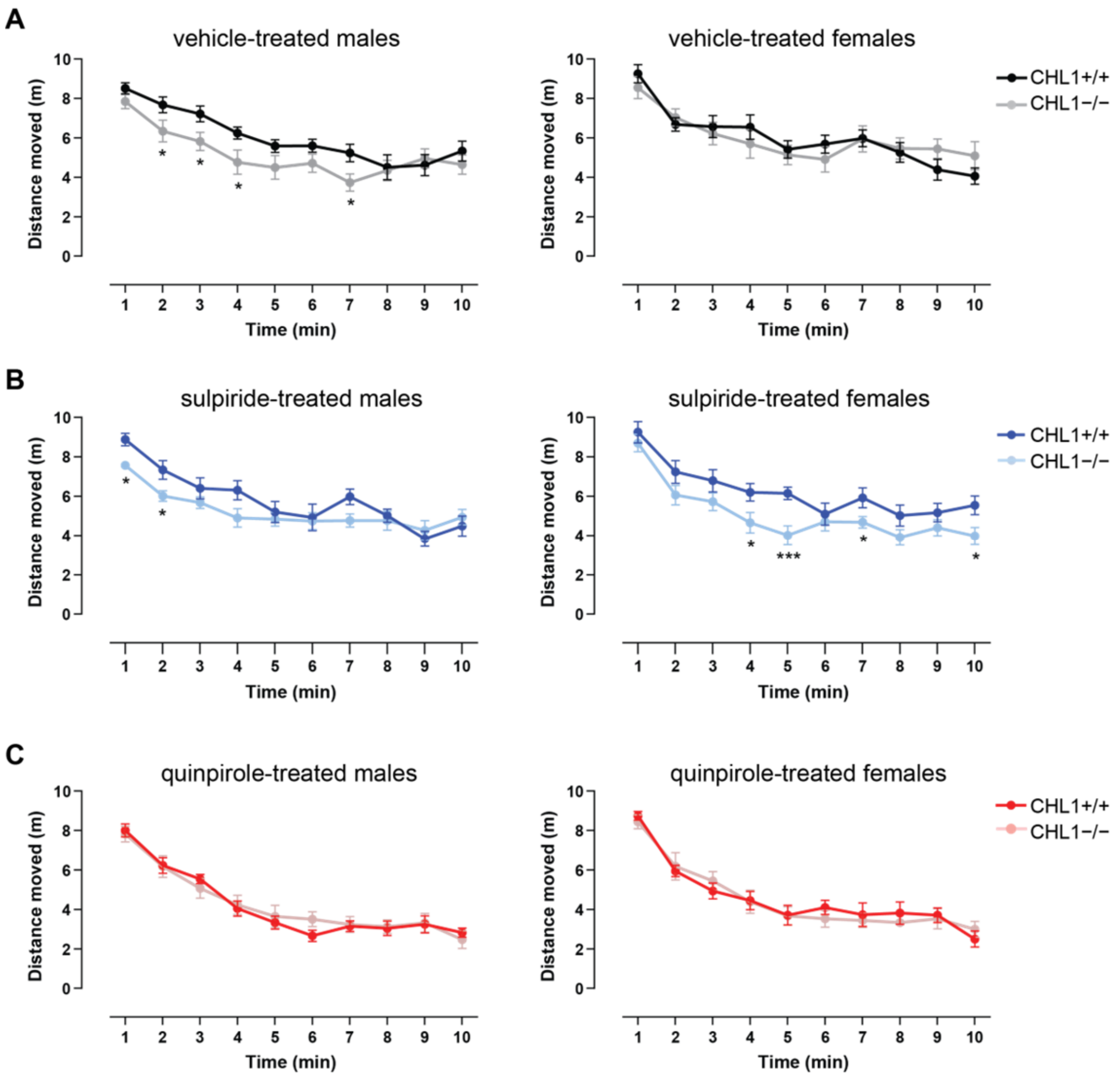

Supplementary Figure S2. Locomotor activity of CHL1+/+ males is reduced to levels of CHL1−/− males by quinpirole treatment, while CHL1−/− females move less after sulpiride treatment. Three-month-old CHL1+/+ and CHL1−/− males and females were treated with vehicle (**A**), sulpiride (**B**) or quinpirole (**C**), and activity in the open field was recorded for 10 min. Total distance moved was determined in 1 min time bins. Values are presented as mean ± SEM (n = 11-13 mice per group) and were analyzed with three-way repeated measures ANOVA followed by the Bonferroni correction post-hoc test (*$p < 0.05$, ***$p < 0.001$, statistical difference from vehicle-treated correspondent genotype).

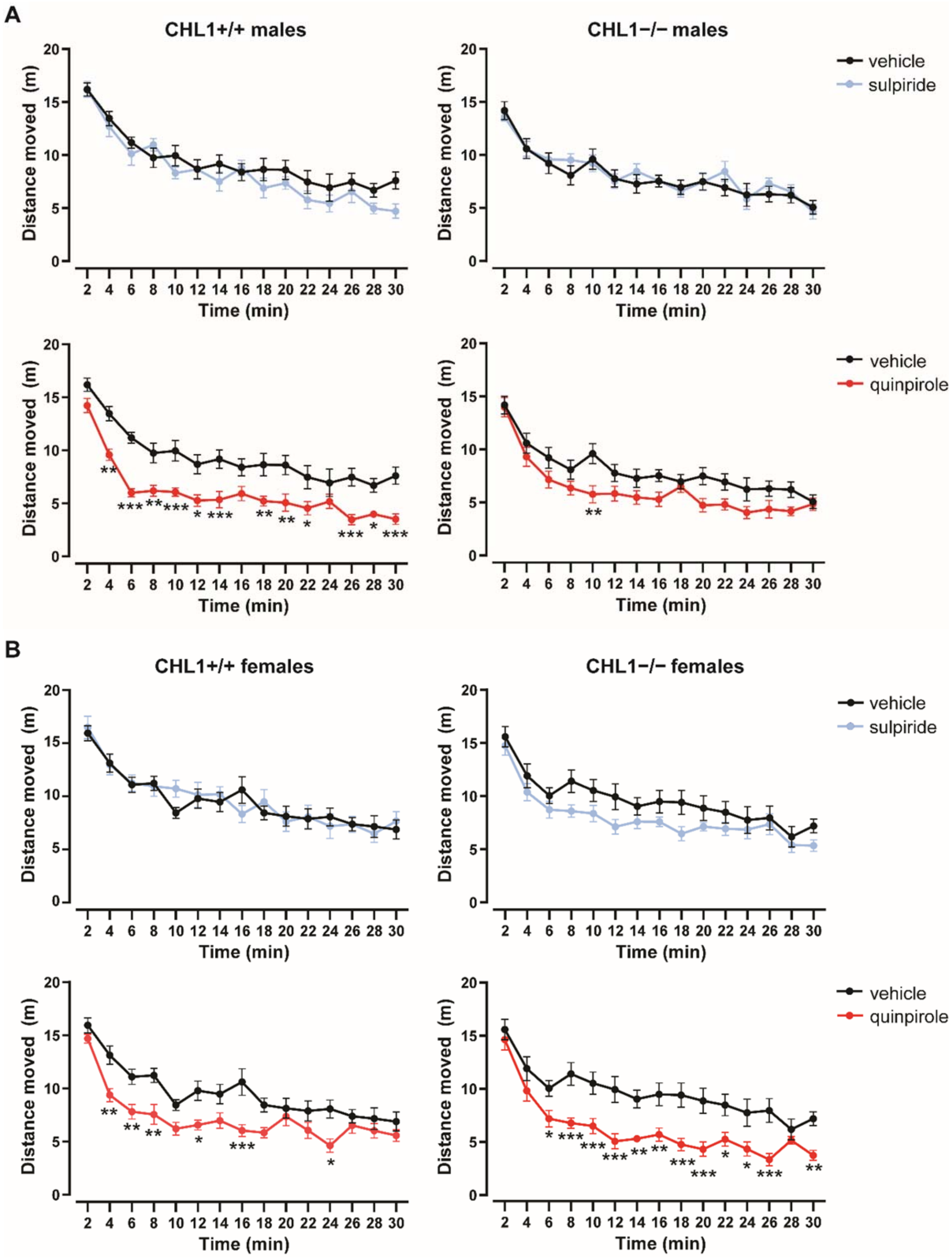

Supplementary Figure S3. Delayed reduction of locomotor activity of CHL1−/− males and females by quinpirole treatment. Three-month-old CHL1+/+ and CHL1−/− males (**A**) and females (**B**) were treated with vehicle, sulpiride or quinpirole, and activity in the open field was recorded for 30 min.

Total distance moved was determined in 2 min time bins. Values are presented as mean ± SEM (n = 11-13 mice per group) and were analyzed with three-way repeated measures ANOVA followed by the Bonferroni correction post-hoc test (*$p < 0.05$, **$p < 0.005$, ***$p < 0.001$, statistical difference from vehicle-treated correspondent genotype).

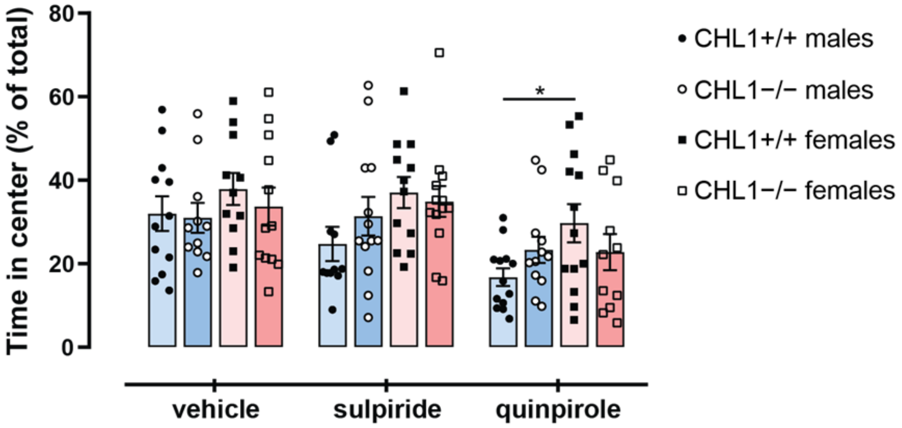

Supplementary Figure S4. CHL1+/+ and CHL1−/− mice spent similar times in the center of the open field. Three-month-old CHL1+/+ and CHL1−/− males and females were treated with vehicle, sulpiride or quinpirole, and percentage of time spent in the center of the open field was calculated. Values are presented as single values and mean ± SEM (n = 11-13 mice per group) and were analyzed with three-way ANOVA followed by the Bonferroni correction post-hoc test (*$p < 0.05$, statistical difference between sex). Blue bars: male mice, magenta bars: female mice.

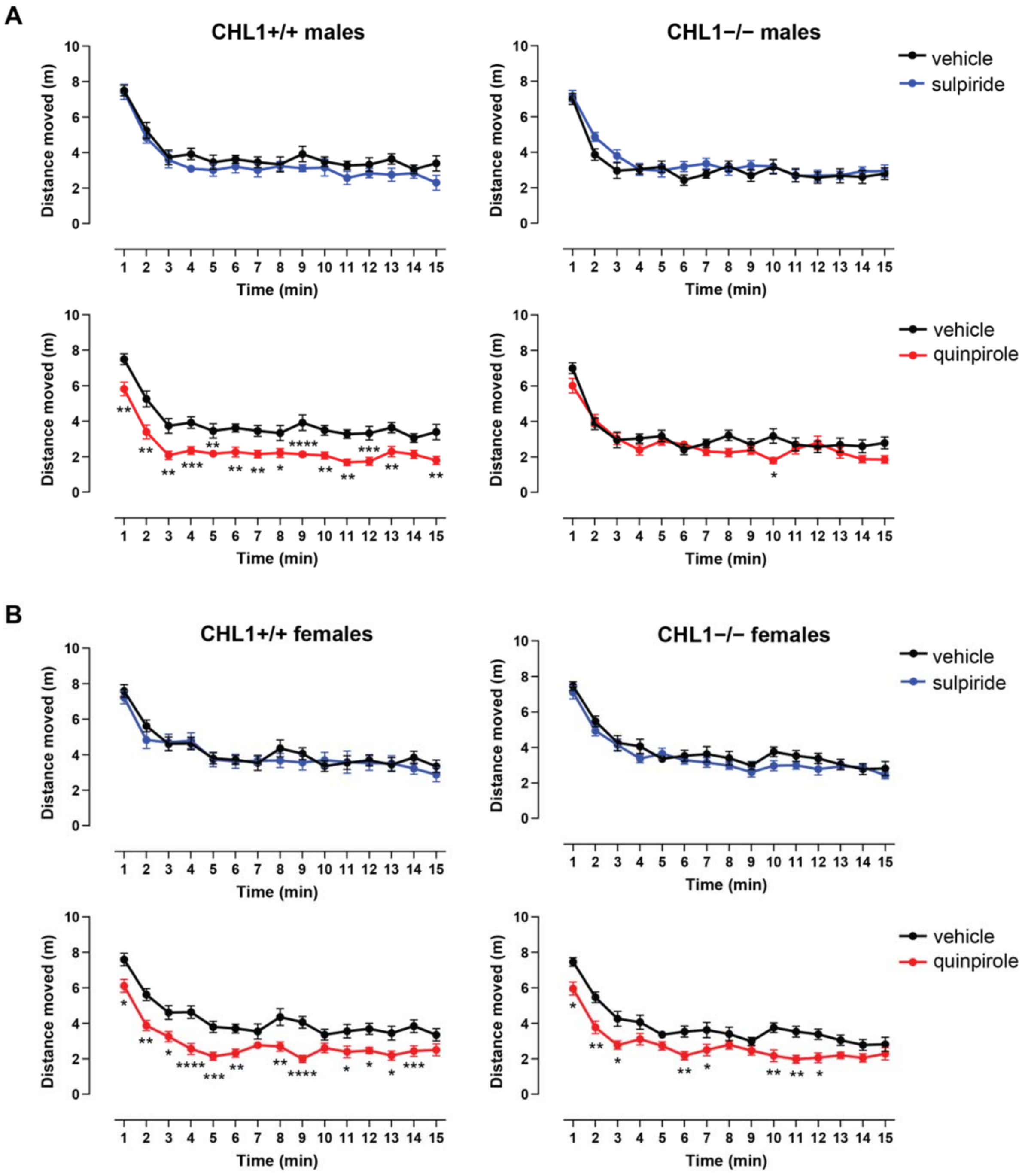

Supplementary Figure S5. Reduced locomotor activity of mice in the Y-maze after quinpirole treatment. Three-month-old CHL1+/+ and CHL1−/− males and females were treated with vehicle, sulpiride or quinpirole, and activity in the Y-maze was recorded for 15 min. Total distance moved of male (**A**) and female (**B**) CHL1+/+ and CHL1−/− mice was determined in 1 min time bins. Values are presented as mean ± SEM (n = 11-13 mice per group) and were analyzed with three-way repeated measures ANOVA followed by the Bonferroni correction post-hoc test (*p < 0.05, **p < 0.01, ***p < 0.001, ****p < 0.0001 statistical difference from vehicle-treated correspondent genotype).

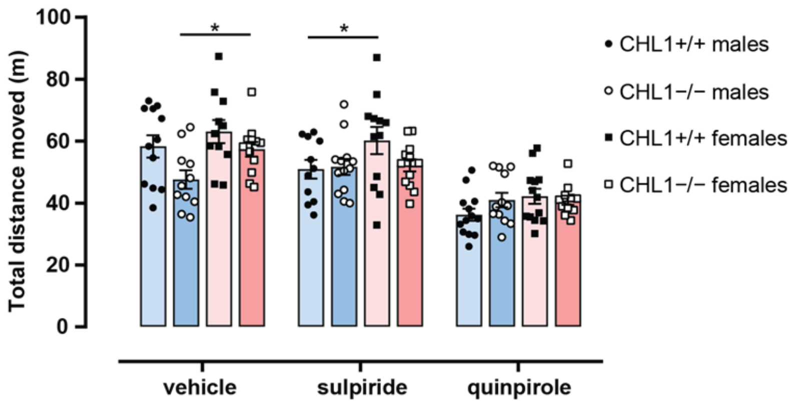

Supplementary Figure S6. Differences in locomotor activity of male and female mice in the Y-maze. Three-month-old male and female CHL1+/+ and CHL1−/− mice were treated with vehicle, sulpiride or quinpirole, and activity in the Y-maze was recorded for 15 min. Total distance moved of mice was determined for 15 min. The 15 min values are shown as single values and mean ± SEM (n = 11-13 mice per group) and were analyzed with three-way ANOVA followed by the Bonferroni correction post-hoc test (*p < 0.05, statistical difference from mice with opposite sex with correspondent treatment). Blue bars: male mice, magenta bars: female mice.

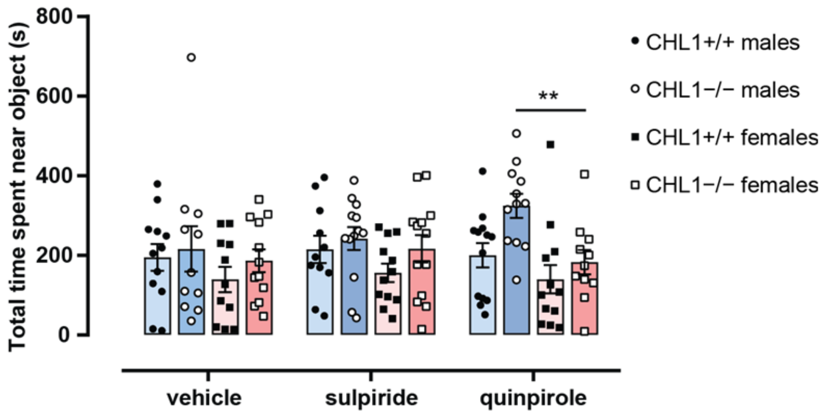

Supplementary Figure S7. Quinpirole-treated CHL1−/− males spend more time near the novel object compared to CHL1−/− females. Four-month-old male and female CHL1+/+ and CHL1−/− mice were treated with vehicle, sulpiride or quinpirole, and activity in the novel object test was recorded for 20 min. The time spent near the novel object was determined. The 20 min values are presented as single values and mean ± SEM (n = 11-13 mice per group) and were analyzed with three-way ANOVA followed by the Bonferroni correction post-hoc test (**p < 0.01, difference between sex). Blue bars: male mice, magenta bars: female mice.

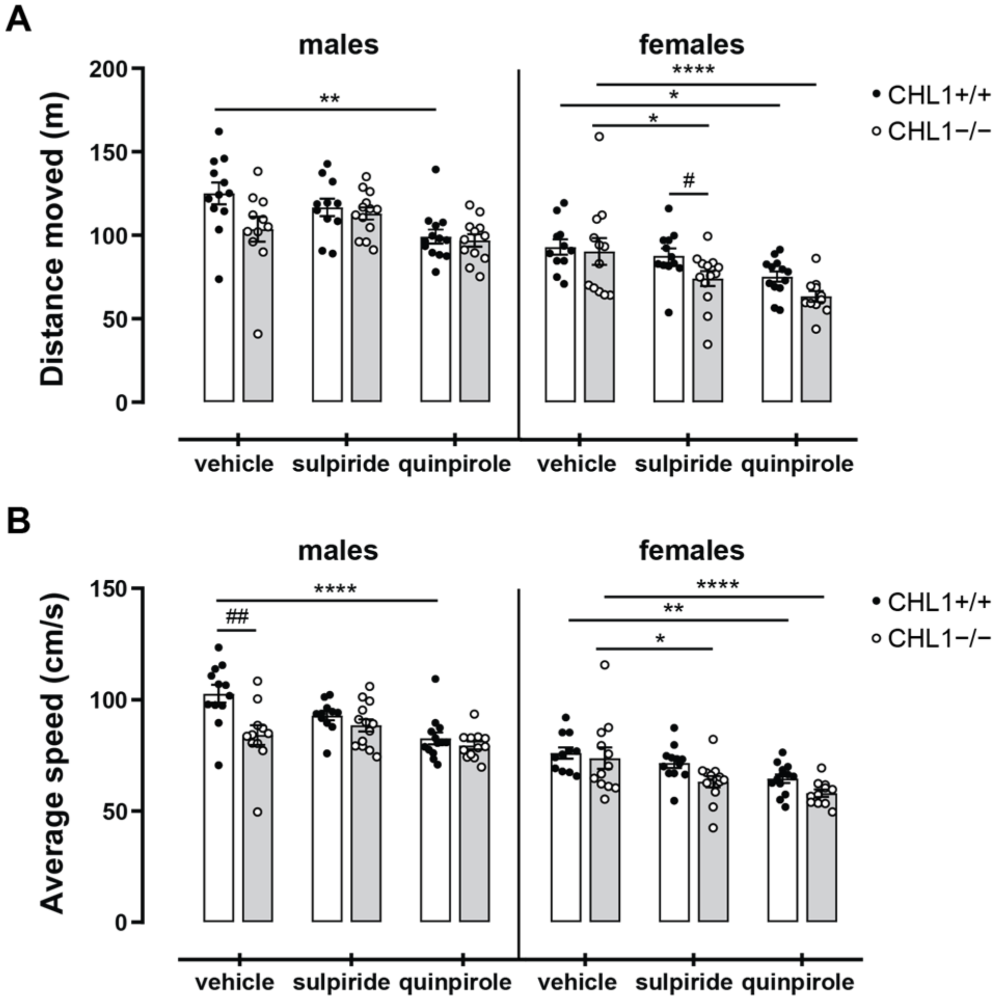

Supplementary Figure S8. Reduced locomotor activity of male CHL1−/− mice and quinpirole-induced hypolocomotion of CHL1+/+ males and CHL1+/+ and CHL1−/− females. Four-month-old male and female CHL1+/+ and CHL1−/− mice were treated with vehicle, sulpiride or quinpirole, and activity in the novel object test was recorded for 20 min. Distance moved in the arena (**A**) and average speed (**B**) during the 20 min trial were determined. Values are presented as single values and mean ± SEM (n = 11-13 mice per group) and were analyzed with three-way ANOVA followed by the Bonferroni correction post-hoc test (*p < 0.05, **p < 0.01, ****p < 0.0001 statistical difference from vehicle-treated correspondent genotype; $^{\#}$p < 0.05, $^{\#\#}$p < 0.01 difference between genotypes). White bars: CHL1+/+ mice, grey bars: CHL1−/− mice.

2.2 Supplementary Tables

Supplementary Table 1. Summary of ANOVA results for all analyzed parameters shown in the main figures. Significant ANOVA results for genotype (G), treatment (T), and sex (S) factors are presented, and both main effects and interaction terms (*) are indicated. P-values are presented as the entire value; a p-value (p) > 0.05 was considered statistically not significant (n.s.) and p-values < 0.05 were considered statistically significant.

Figure	Statistical test	Significant ANOVA factors or interactions
**Figure 2**		
(A) Total distance moved (m) (10 min)	Three-way ANOVA	G: F(1,132) = 6.814, p = 0.0100<br>T: F(2,132) = 26.734, p = 0.0001
(B) Total distance moved (m) (30 min)	Three-way ANOVA	G: F(1,132) = 7.240, p = 0.0080<br>T: F(2,132) = 44.277, p = 0.0001<br>S: F(1,132) = 7.038, p = 0.0090<br>G*T*S: F(2,132)=3.417 p=0.0360
(C) Average speed (cm/s)	Three-way ANOVA	G: F(1,132) = 9.891, p = 0.0020<br>T: F(2,132) = 45.717, p = 0.0001<br>S: F(1,132) = 8.391, p = 0.0040<br>G*T*S: F(2,132)=3.243 p=0.0420
**Figure 3**		
(A) and (B) Distance moved (m)	Three-way repeated measures ANOVA	G: F(1,132) = 6.814, p = 0.0100<br>T: F(2,132) = 26.734, p = 0.0001
**Figure 4**		
(A) Total distance moved (m)	Three-way ANOVA	same as Fig. 2B
(B) Distance moved in center (m)	Three-way ANOVA	T: F(2,132) = 21.877, p = 0.0001<br>G*S: F(1,132) = 3.382, n.s.
(C) Grooming time (s)	Three-way ANOVA	T: F(2,132) = 4.470, p = 0.0130<br>S: F(1,132) = 9.731, p = 0.0020
**Figure 5**		
(A) Time moving in center (%)	Three-way ANOVA	T: F(2,132) = 6.821, p = 0.0020<br>S: F(1,132) = 4.127, p = 0.0440<br>G*S: F(1,132) = 4.493, p = 0.0360
(B) Time in center (s)	Three-way ANOVA	n.s.
(C) Total distance moved in the center (m)	Three-way ANOVA	same as Fig. 4B
(D) Average distance to wall (cm)	Three-way ANOVA	G: F(1,132) = 6.524, p = 0.0012
**Figure 6**		
(A) Grooming latency (s)	Three-way ANOVA	S: F(1,132) = 7.487, p = 0.0070<br>G*T: F(2,132) = 3.386, p = 0.0370

(B) Grooming time (s)	Three-way ANOVA	same as Fig. 4C
(C) Unsupported rearing (n)	Three-way ANOVA	G: F(1,132) = 15.118, p = 0.0001
(D) Supported rearing (n)	Three-way ANOVA	G: F(1,132) = 7.393, p = 0.0070<br>T: F(2,132) = 7.782, p = 0.0010
(E) Fecal boli number	Three-way ANOVA	T: F(2,132) = 8.646, p = 0.0001<br>S: F(1,132) = 12.457, p = 0.0010
(F) Wall jumping (n)	Three-way ANOVA	G: F(1,132) = 8.618, p = 0.0040
**Figure 7**		
(A) Number of correct alternations (out of 24)	Three-way ANOVA	G: F(1,131) = 10.960, p = 0.0010
(B) Time to complete 24 alternations (min)	Brown-Forsythe ANOVA	Males: F(5,57.705) = 8.992, p = 0.0001<br>Females: F(5,50.028) = 15.460, p = 0.0001
**Figure 8**		
(A) Time spent at novel object (s)	Three-way ANOVA	G: F(1,132) = 7.625, p = 0.0070<br>T: F(2,132) = 0.763, n.s.S: F(1,132) = 10.057, p = 0.0020
(B) Distance moved near object (m)	Three-way ANOVA split treatment factor (vehicle-sulpiride or vehicle-quinpirole)	Sulpiride: n.s.<br>Quinpirole: S: F(1,87) = 9.165, p = 0.0030<br>G*T: F(1,87) = 3.255, n.s. T*S: F(1,87) = 3.277, n.s.
(C) Latency to reach object (s)	Three-way ANOVA	n.s.
**Figure 9**		
Preference index (%)	Two-way ANOVA split sex factor	Males: n.s.<br>Females: n.s.
**Figure 10**		
(A) Frequency unfamiliar (n)	Two-way ANOVA split sex factor	Males: T: F (2,66) = 3.466, p = 0.0370<br>Females: n.s.
(B) Frequency familiar (n)	Two-way ANOVA split sex factor	Males: T: F (2,66) = 4.859, p = 0.0108<br>Females: T: F (2,66) = 6.755, p = 0.0021

Supplementary Table 2. Summary of ANOVA results for all analyzed parameters shown in the supplementary figures. Significant ANOVA results for genotype (G), treatment (T), and sex (S) factors are presented, and both main effects and interaction terms (*) are indicated. P-values are presented as the entire value; a p-value (p) > 0.05 was considered statistically not significant (n.s.) and p-values < 0.05 were considered statistically significant.

Figure	Statistical test	Significant ANOVA factors or interactions
**Suppl. Figure 2**		
(A), (B) and (C) Distance moved (m)	Three-way repeated measures ANOVA	see Fig. 3
**Suppl. Figure 3**		
(A) and (B) Distance moved (m)	Three-way repeated measures ANOVA	same as Fig. 2B
**Suppl. Figure 4**		
% of time in the center	Three-way ANOVA	S: F(1,132) = 4.369, p = 0.0390
**Suppl. Figure 5**		
(A) and (B) Distance moved (m)	Three-way repeated measures ANOVA	G: F(1,132) = 4.260, p = 0.0410<br>T: F(2,132) = 39.211, p = 0.0001<br>S: F(1,132) = 9.466, p = 0.0030<br>G*T: F(2,132) = 3.085, p = 0.0490
**Suppl. Figure 6**		
Total distance moved (m)	Three-way ANOVA	see Suppl. Fig. 5
**Suppl. Figure 7**		
Total time spent near object (s)	Three-way ANOVA	see Fig. 8A
**Suppl. Figure 8**		
(A) Distance moved (m)	Three-way ANOVA	G: F(1,131) = 8.814, p = 0.0040<br>T: F(2,131) = 19.030, p = 0.0001<br>S: F(1,131) = 8.500, p = 0.0040
(B) Average speed (cm/s)	Three-way ANOVA	G: F(1,131) = 15.474, p = 0.0001<br>T: F(2,131) = 22.784, p = 0.0001

References

Castro, S. W., and Strange, P. G. (1993). Differences in the ligand binding properties of the short and long versions of the D2 dopamine receptor. *J. Neurochem*. 60, 372-375. doi: 10.1111/j.1471-4159.1993.tb05863.x

Chari, T., Griswold, S., Andrews, N. A., and Fagiolini, M. (2020). The stage of the estrus cycle is critical for interpretation of female mouse social interaction behavior. *Front. Behav. Neurosci*. 14, 113. doi: 10.3389/fnbeh.2020.00113

McClintock, M. K. (1978). Estrous synchrony and its mediation by airborne chemical communication (Rattus norvegicus). *Horm. Behav*. 10, 264-275. doi: 10.1016/0018-506x(78)90071-5

Meziane, H., Ouagazzal, A. M., Aubert, L., Wietrzych, M., and Krezel, W. (2007). Estrous cycle effects on behavior of C57BL/6J and BALB/cByJ female mice: implications for phenotyping strategies. *Genes Brain Behav*. 6, 192-200. doi: 10.1111/j.1601-183X.2006.00249.x

Vallone, D., Picetti, R., and Borrelli, E. (2000). Structure and function of dopamine receptors. *Neurosci. Biobehav. Rev*. 24, 125–132. doi: 10.1016/s0149-7634(99)00063-9
